# RAMZIS: a bioinformatic toolkit for rigorous assessment of the alterations to glycoprotein composition that occur during biological processes

**DOI:** 10.1093/bioadv/vbae012

**Published:** 2024-01-25

**Authors:** William Edwin Hackett, Deborah Chang, Luis Carvalho, Joseph Zaia

**Affiliations:** Bioinformatics Program, Boston University, Boston, MA 02215, United States; Department of Biochemistry, Boston University, Boston, MA 02215, United States; Bioinformatics Program, Boston University, Boston, MA 02215, United States; Department of Mathematics, Boston University, Boston, MA 02215, United States; Bioinformatics Program, Boston University, Boston, MA 02215, United States; Department of Biochemistry, Boston University, Boston, MA 02215, United States

## Abstract

**Motivation:**

Glycosylation elaborates the structures and functions of glycoproteins; glycoproteins are common post-translationally modified proteins and are heterogeneous and non-deterministically synthesized as an evolutionarily driven mechanism that elaborates the functions of glycosylated gene products. Glycoproteins, accounting for approximately half of all proteins, require specialized proteomics data analysis methods due to micro- and macro-heterogeneities as a given glycosite can be divided into several glycosylated forms, each of which must be quantified. Sampling of heterogeneous glycopeptides is limited by mass spectrometer speed and sensitivity, resulting in missing values. In conjunction with the low sample size inherent to glycoproteomics, a specialized toolset is needed to determine if observed changes in glycopeptide abundances are biologically significant or due to data quality limitations.

**Results:**

We developed an R package, Relative Assessment of *m/z* Identifications by Similarity (RAMZIS), that uses similarity metrics to guide researchers to a more rigorous interpretation of glycoproteomics data. RAMZIS uses a permutation test to generate contextual similarity, which assesses the quality of mass spectral data and outputs a graphical demonstration of the likelihood of finding biologically significant differences in glycosylation abundance datasets. Investigators can assess dataset quality, holistically differentiate glycosites, and identify which glycopeptides are responsible for glycosylation pattern change. RAMZIS is validated by theoretical cases and a proof-of-concept application. RAMZIS enables comparison between datasets too stochastic, small, or sparse for interpolation while acknowledging these issues in its assessment. Using this tool, researchers will be able to rigorously define the role of glycosylation and the changes that occur during biological processes.

**Availability and implementation:**

https://github.com/WillHackett22/RAMZIS.

## 1 Introduction

### 1.1 The difficulties of quantitative glycoproteomics

Evolutionary pressure for multicellularity and pathogen defense drives the complexity of protein glycosylation (Dennis 2017). Mammalian glycoprotein glycosites are modified with a distribution of glycoforms resulting from the non-template driven ER-Golgi biosynthesis. Glycosylation modulates protein physicochemical properties and the interactions with protein binding partners, and each glycosylation site on a protein contributes to these changes. Understanding the biological functions of individual glycoproteins requires assessing the quantitative changes to protein site glycosylation patterns. Herein, we primarily focus on N-glycosylation composition analysis; N-glycans are a specific type of glycan that attach to asparagines given specific surrounding amino acid motifs—glycosites (Stanley *et al.* 2022).

High-throughput glycosylation is quantified via mass spectrometry, and the experimental methods involved have many inherently limiting factors that require nuanced analysis. Glycopeptides are peptide backbones with specific attached glycans; they are more numerous, have lower signal, and are more complicated to identify via scoring than their deglycosylated peptide counterparts. These factors, in addition to several others, result in lower confidence identifications, more variable quantifications, and a high degree of missingness. These problems are further compounded by the low sample size inherent to glycoproteomics mass spectrometry experiments.

Liquid chromatography tandem mass spectrometry (LC-MSMS) works by the elution of ions from a liquid chromatography column into a mass spectrometer, wherein these precursor ions have their mass-to-charge ratio (*m*/*z*) measured before undergoing dissociation to produce fragment or product ions, which are similarly measured; these product ions are then scored to determine the identity of their associated precursor (Zhu *et al.* 2017, Stanley *et al.* 2022). Mass spectrometers have maximal throughputs for the number of precursor ions that can be acquired for fragmentation in a given time period. Longer, slower elutions allow LC-MSMS experiments to acquire more ions and score them more confidently, but machine time is limited and longer elutions means fewer samples may be run (Yang *et al.* 2017, Zhu *et al.* 2017, Hackett and Zaia 2021).

This conflict between precursor throughput and sample size is exaggerated in glycoproteomic LC-MSMS experiments, where for an individual glycosite there are hundreds of viable glycans that could be observed, multiplying the number of potential precursors. This is further exacerbated in quantification experiments wherein the same precursor must be identified multiple times in the same elution as glycopeptide abundance is measured by taking the area under the curve (AUC) of the elution. Different acquisition methods—strategies used by the mass spectrometer in the acquisition and fragmentation of precursors—can help to mitigate some of these issues, but there is always a trade-off. Data Dependent Acquisition (DDA) worsens the missing value problem by failing to sample lower abundance, co-eluting glycopeptides (Chang *et al.* 2021, Ye and Vakhrushev 2021). Meanwhile, Data Independent Acquisition does sample all precursor ions, but its more limited sensitivity and higher incidence of coisolation produces more uniform missing values and lower confidence identifications and more variable quantifications (Chang *et al.* 2021, 2022). Were these issues alone not bad enough, quantitative glycoproteomics must also contend with a host of other issues, such as variability introduced by enrichment protocols, identification inconsistency stemming from ionization energy and search space optimization issues, and signal splitting across different glycoforms of the same composition. There is no silver bullet solution to glycoproteomics woes. As a result of these factors and others, the ability to quantify changes in glycoprotein glycosylation is limited.

LC-MSMS is still the best high-throughput method available for glycopeptide identification and quantification. A given glycosite elutes over a narrow time period, and glycopeptides from the same glycosite can be ambiguous due to the combinatorial nature of glycans and the similar masses of glycan subunits. Confident identification and quantification of glycopeptide glycoform compositions requires tandem MS acquisition of the glycopeptide precursor ions (Hinneburg *et al*. 2016, Riley *et al.* 2020). The study of quantitative glycosylation pattern changes of individual glycosite compositions, let alone glycoprotein wide structural changes, has many barriers to overcome.

However, the study of N-glycosylation cannot simply be abandoned until such time as these barriers are overcome. As shown by numerous recent publications and preprints concerning the SARS-CoV-2 spike protein (Casalino *et al.* 2020, Shajahan *et al*. 2020, Watanabe *et al.* 2020, Zhang *et al*. 2021, Chang *et al.* 2021, Sanda *et al.* 2021), there are many biomedical laboratories with the capability to assign site-specific glycosylation for highly complex glycoproteins using DDA LC-MSMS datasets. In order to use these data effectively, we cannot analyze it by conventional statistical approaches; it is too variable with too much missingness and too little sample size.

Quantitative glycoproteomic data need a tailored analysis. For any analysis to be trusted, it must include an assessment of the data quality before it ever assesses changes in glycosylation. Given the inherent limitations of the data, it is prudent to be conservative and look at changes across a glycosylation site, rather than target-specific glycopeptides. To address this, we developed RAMZIS, a guiding toolset using a similarity metric in a permutation test that assesses N-glycosylated glycosites for their data quality, identifies potential outlier samples, determines a likelihood for differentiating glycosylation patterns, and ranks the contribution of glycopeptides to that likelihood. This tool can be used for N- and O- glycosylation data, as well as phosphorylation and other PTMs, but has only been tested on N-glycosylation data in Influenza, SARS-CoV-2, and Glioblastoma (Chang *et al.* 2021, Sethi *et al.* 2022).

### 1.2 Statistical context of glycoproteins

Conventional statistics, such as *t*-tests and ANOVAs, are unlikely to provide accurate results in glycoproteomics datasets as they rely on a greater number of replicates, a lower degree of missingness, and an assumption of relative independence between variables. As mentioned above, none of these factors holds true for glycopeptide abundance data. Glycopeptides are not independent from one another via the biosynthetic network; their log abundances while normal at the global level, are not provably normal at an individual level due to sample size; and they have a high degree of missingness that cannot be imputed away due to sample size issues.

Since there are so many individual glycopeptides, even if conventional tests were performed, the increased risk of false positive comparisons makes standard parametric testing unwise. Many parametric tests discard quantification information in favor of ranking, which reduces our ability to account for effect sizes and has lower power than is acceptable for our typical sample sizes. Fortunately, datasets may be compared collectively, trading specificity for power. Similarity measures indicate how much two things resemble one another and are often used in clustering algorithms and sequence alignment (Pearson, 2021, Stanley *et al.* 2022).

The Tanimoto similarity metric is a derivation of the more common Jaccard Index, which uses binary categorical information to determine similarity between two subsets by dividing the intersection of the subsets by their union. The Tanimoto compares vectors of two continuous data points, operating off the same general principle of the Jaccard, to create a similarity scale from zero, not at all similar, to one, practically identical. Chemoinformatics, the joint field of computer science applied to physical chemistry, makes use of them in molecular fingerprinting, a tool for drug screening and chemical mapping. The Tanimoto similarity metric can be used to identify a molecule by comparison to a database based on a set of its chemical and physical attributes (Bajusz *et al.* 2015). The Tanimoto coefficient has been used to evaluate biological species co-occurrences based on presence/absence data to elucidate relationships among organisms (Chung *et al.* 2019). Glycoproteomics has even made use of it before in GproDIA, which uses it for spectral scoring (Yang *et al.* 2021).

Glycoproteomic relative abundances can be grouped by glycosite and compared using this transformation, but additional scaling factors should be introduced to account for the missingness in glycoproteomics data and its adverse influence on reliability. The Tanimoto similarity metric was weighted for this purpose. To understand what the similarity meant, we created contextual similarity- context generated via a permutation test of this weighted similarity metric. Permutation tests resample data to simulate comparisons between the same datasets and have been found to be effective above the thresholds used here (Noguchi *et al.* 2021).

This contextual similarity allows for the above-mentioned assessments of quality and pattern differentiation via four types of comparison. First, the quality of a dataset and outlier identification is observed through a Self or Internal comparison. These comparisons test how similar samplings of a dataset are to other samplings, and they are most heavily affected by missingness, enforcing a conservative approach to data quality.

Second, the desired comparisons of control versus variant (mutant or disease state), here referred to as Test comparisons, comes from comparing the samplings of the datasets against samplings of the other, showing the distribution of how much the datasets look like one another. The Internal similarities use the Test similarity distribution as a reference for their quality assessment.

The third context is a null hypothesis, comparisons made as if there were no difference between the two samples. This Null Similarity is simulated by comparing random samplings of a joint dataset; this shows the similarity behavior wherein glycopeptides from both sample datasets were sampled from the same underlying distribution. Combined with the Test comparisons, the Null hypothesis indicates the likelihood of separable, differentiable glycosites, and informs on the viability of the comparison.

The final context is the unsampled, direct comparison of the two datasets: the Actual Similarity, which is a single point that is used to assess the simulation validity of the Test Similarity.

After providing a general assessment of viability, we then rank individual glycopeptides for follow up targeted experiments or more conventional statistical analyses. Here, we describe the RAMZIS internal algorithm and criterion and its application to re-analysis of published data, reconfirming prior work, and expanding on these analyses.

## 2 Methods

RAMZIS is an R package that takes the abundances of two sample group glycopeptides as input and produces a data quality assessment and visualization, a general comparison with a visualization, and a ranked list of glycopeptides as output. It does this via contextual similarity, whereby related similarity comparisons made with a modified Tanimoto similarity metric provide context for one another as a form of permutation test. The overall algorithm is illustrated in Supplementary Fig. S1.

### 2.1 Modified Tanimoto metric

The Tanimoto similarity metric was modified in order to increase our ability to differentiate glycoprotein glycosites based on abundances of glycopeptides. The traditional metric is the sum of sample group A times sample group B scaled to the contributions of A and B as follows:

(1) $$T_{\mathrm{Original}}=\sum_{i}^{n}\frac{A_{i}B_{i}}{\sum_{i}^{n}A_{i}^{2}+\sum_{i}^{n}B_{i}^{2}-\sum_{i}^{n}A_{i}B_{i}}.$$

We modified the metric to include both the presence and abundance of glycopeptide glycoforms as follows:

(2) $$T_{\mathrm{Mod}}=\sum_{i}^{n}\frac{A_{i}P_{i,A}B_{i}P_{i,B}K^{-d(A_{i}B_{i})}}{\sum_{i}^{n}(A_{i}P_{i,A}{)}^{2}+\sum_{i}^{n}(B_{i}P_{i,B}{)}^{2}-\sum_{i}^{n}A_{i}P_{i,A}B_{i}P_{i,B}K^{-d(A_{i}B_{i})}}.$$

Where *i* is an identification; *n* is the number of identifications in a subset or glycosite; *A* and *B* refer to sample groups, meaning *Ai* refers to the relative log abundance of glycopeptide *i* in group *A*; *Pi*, *A* refers to the presence of glycopeptide *i* in group *A*; *K* is a distance scaling term equal to one plus the mean presence rate of glycopeptide *i* in both groups; *d*(*Ai*, *Bi*) is the Manhattan distance between both groups at glycopeptide *i*.

These modifications were developed by observing the behavior of real and simulated data. The presence term impacts Internal Similarity more than any other context; by allowing high missingness to negatively impact similarity, the Internal similarities become more likely to overlap with the less affected Test similarity, making data quality rejection more likely. This promotes a conservative data quality assessment. The distance scaling term helps to weight reliable differences; glycopeptides that are fully observed in both datasets are allowed to be considered more dissimilar, and this weighting is done proportionate to distance to encourage the identification of larger differences when testing. The Null similarity has a higher average presence than the Test similarity, while large differences are more likely in the Test than the Null. These factors mean that on average, the Null has more glycopeptides weighted to be dissimilar, except in cases of large, consistently quantified glycopeptides in the Test. This promotes the identification of larger, more reliable differences in glycosylation patterns.

### 2.2 RAMZIS workflow

#### 2.2.1 Step 1: Data format and standardization

The input for RAMZIS is two matrices of the relative abundances of identified glycopeptides by sample; it can delineate between exact peptide matches, and glycosite notations can be given to subset the data more comprehensively.

RAMZIS scales signal strength through relative log abundance and can also scale in proportion to the log of the provided TIC of each replicate. This ensures that variances in signal strength and identification rate outliers are more easily identified. A minimum number of observations per identification is used to pre-emptively filter poor quality results, which can be disabled as desired.

#### 2.2.2 Step 2: Bootstrapping datasets

As described earlier replicates were sampled with replacement to produce three kinds of collections of datasets that were then used to produce four similarity distributions. A collection herein refers to the set of all sampled datasets of a specific type.

A test collection is composed of multiple test datasets, which are each made solely from one of the sample groups with as many replicates as the observed sample group. This is done for both sample groups, making two test collections. For each test collection, 100 samplings are created prior to duplicate reduction. The test collections are used to generate two Internal Similarity Distributions and the Test Similarity Distribution.

A null collection is made of datasets that sample from both sample groups with replicates equal to the number of replicates seen in the original sample groups. Sampling is done at a roughly equal rate from both groups to prevent outlier effects; duplicate datasets are eliminated from the collection to prevent spurious outlier effects in the similarity comparisons. For each null collection, 200 datasets are generated prior to duplicate reduction.

#### 2.2.3 Step 3: Similarity distributions and observed similarity

Four kinds of similarity comparison are made. One is the test collection comparison, which calculates the similarity between every sampling from one test collection to every sampling of the other test collection; this creates a maximum of 10 000 test comparisons. These test comparisons make up the Test Similarity Distribution; it simulates the extent to which the two sample groups resemble one another.

The second kind has two samplings per analysis, and they are the internal comparisons, which compare samplings of a sample group’s test collection against the rest of that group’s test collection. This generates a maximum of 4950 internal comparisons—the maximum number of unique comparisons with 100 samplings—limited to prevent combinatorial expansion in larger datasets. These distributions are the Internal Similarity Distributions for their respective sample groups; they represent the extent to which a sample group resembles itself and provide a check on data quality through internal consistency.

The third group of similarity comparisons are null comparisons. These work like the test comparisons. They produce a maximum of 40 000 comparisons—the maximum number of unique comparisons with 200 samplings per sample group—limited to prevent combinatorial expansion and reduce computation needed. This distribution of comparisons is the Null Similarity Distribution; it represents how much a random comparison within the dataset resembles any other random comparison within the dataset. It is used as a reference point for the Test Similarity Distribution.

The final similarity comparison grouping is called the Observed Similarity Comparison and is the singular comparison between the observed sample groups from the original data. This is the similarity of the data without bootstrapping and is a key reference value in evaluating simulation quality, data quality, and glycopeptide ranking.

#### 2.2.4 Step 4: General data quality assessment

The extent to which similarity comparisons can be made meaningfully is influenced by the quality of the LC-MS data ion abundances and reproducibility. The various similarity distributions allow for assessing this quality through their means, variances, and modalities.

The first assessment uses the two Internal Distributions. This is done to determine if there are outliers in the data via modality and to determine the reliability of the data via Test Distribution overlap.

Outlier detection is performed by examining the peak membership of multimodal Internal Distributions. Peak membership is the proportion of replicates that contribute to comparisons within a defined peak range; non-uniform peak membership by an individual replicate indicates an outlier. Deviation from uniform distribution is determined via a *z*-score, where the replicate in question is withheld from the mean and standard deviation calculations. If the *z*-score exceeds three—indicating that the chance the replicate is from the same underlying normal distribution is <1%—for a sample in a peak then it is marked as being either over or under-represented within that peak; it should only be removed if it is under-represented in the primary peak or overrepresented in a non-primary peak, and if that peak contains more than 10% of the overall comparisons.

RAMZIS is currently unable to use this outlier detection method for subgroup identification due to the number of subset comparisons required to test this. If the distributions are still multimodal after performing replicate removal, then there is either an underlying data quality problem or a subset within the sample group; either will prevent appropriate comparison in the given dataset.

Data reliability is assessed via confidence scores called the Internal Confidence (IC); these ICs are derived by the relation of the Internal Distributions to the Test Distribution. The IC of each Internal Distribution is a weighted *z*-score defined in (5); the score is the absolute distance between the means (*µ*) of the Internal and Test Distributions, divided by the standard deviation (*σ*) of the Internal Distribution, and then scaled according to the degree of overlap between the two distributions. The overlap scaling term is the sum of the false positive rate (FPR), alpha, and the false negative rate (FNR), beta. Alpha and beta overlaps are further explained in the Supplementary Text and generally defined by (3) and (4).

(3) $$\mathrm{AlphaOverlap}\left(T,R\right)=\frac{\int_{x}^{!}T}{T+R-(\int_{x}^{1}T+\int_{0}^{x}R)}.$$

(4) $$\mathrm{BetaOverlap}\left(T,R\right)=\frac{\int_{0}^{x}R}{T+R-(\int_{x}^{1}T+\int_{0}^{x}R)}.$$

Where *T* is the density of the Test Distribution; *R* is the density of the reference similarity distribution, in this case, the Internal Similarities; *x* is the point at which the Test begins to have a lower density than the reference.

(5) $$\mathrm{InternalScore}=\left(\frac{\mu_{\mathrm{Internal}}-\mu_{\mathrm{Test}}}{\sigma_{\mathrm{Internal}}}\right)\times 10^{-\left(\alpha +\beta \right)}.$$

An IC of two indicates less than a 5% chance of overlap between the Internal and Test Distribution means; more conservative users can raise this threshold as they feel appropriate. Internal Distributions will have different IC scores for each comparison they are used in. If the IC is ever negative, this indicates that the Internal Distribution is of lower similarity than the Test Distribution, and that the samples involved may not be replicates. An IC close to zero indicates that the contributing samples may be a subset of the other sample group; if both ICs are close to zero, then all samples likely stem from the same underlying glycosylation pattern.

A prior version of the score was used in a related study (Chang *et al.* 2022); that version used the ratio of the internal peak height to the variance, scaled according to an overlap weighting term. This left out information relating to the distance between Similarity distributions; re-analysis did not substantially change the prior analysis.

The Observed Similarity is used to assess the bootstrapping validity. The Observed Similarity must be within 3 SD of the Test Distribution’s mean for it to be considered reliable. A higher standard can be imposed by decreasing the allowable deviation or by using a central quartile assessment, meaning that it must be within the central 50% of the Test Distribution. If this metric fails, the bootstrapped sampling did not reliably simulate the original comparison of datasets; this failure is more likely to happen in highly similar datasets or in datasets with very low variability.

A final sanity check comes from comparing the Null Distribution to the Test Distribution; if the Null is lower than the Test, it indicates very high degrees of internal variability and low degrees of missingness, which should be identifiable in the Internal Distributions.

#### 2.2.5 Step 5: General comparison

If data quality is acceptable and outlier effects are removed, a general comparison between the Test and Null Distributions can be made. If the two underlying distributions are significantly differentiable then it is likely that the sample groups have differentiable quantification patterns. Differentiability is determined here by overlap proportion as described above in (3) and (4). Conventional standards place differentiability at an alpha, representing FPR, <0.05 and a beta, representing FNR, <0.20.

If alpha and beta are below their thresholds, then the two sample groups are considered differentiable in their overall quantification patterns. The ability to globally differentiate the groups does not guarantee the existence of a consistent differentiable identification between the groups, but it does make it more likely. It is possible that individual assessments will indicate a need for improved data quality rather than be differentiable themselves. This overview tells the user that differentiating the sample groups is possible.

A visual display is generated to assist the user in understanding this comparison. It displays the density of the Test Distribution in red, the density of the Null Distribution in blue, the false positive region in black, and the false negative region in grey. Different examples are presented in Section 3 and Supplementary Sections.

#### 2.2.6 Step 6: Ranking assessment and reporting

To guide analysis and experimental design, individual glycopeptide identifications are ranked based on their contributions to the various similarity distributions. Contribution refers to the amount of similarity an identification adds to the total similarity in each comparison. Individual identifications undergo a quality assessment by their contribution to Internal Similarity by examining peak membership and relative position to their Test Similarity contribution. These assessments are performed as they are for the totaled distributions, but combine alpha and beta due to resolution for a total threshold of 25%. Potential causes of failure are identified and reported alongside the identification rankings.

Rank is correlated to the differentiation between their Null and Test group contributions. Unlike the quality assessment, the contributions used herein only use the numerator term of (2); this is because missing values bias the overall denominator term and more likely to occur in Null type datasets, ironically preventing the identification of missing values as contributors to differences in similarity. For a given identification, these unweighted contributions use the average *z*-score of all Test contributions from the context of the Null distribution. Therefore, the lower the *z*-score, the higher the ranking. Ideal ranked glycopeptides will be <−2 if they are sources of differentiation, but this number may be higher in cases of missing values. Significant positive *z*-scores may indicate an underlying interdependence between datasets.

## 3 Results

To display the workings and utility of RAMZIS for a glycoproteomic researcher and explain the usage, we provide examples and a proof-of-concept re-examination. These come in the form of theoretical abundance data as a guide and exploration of published experimental data. The guide provides a range of plausible scenarios to better illustrate the output and utility of the toolset and consists of Supplementary Figs S2–S10 and Supplementary Tables S2–S4. These theoretical examples also prove that RAMZIS does not show significant differences from increase in variability, and that quality assessment is negatively affected by missingness and variability. The re-examination takes data from a published glycoproteomics study (Khatri *et al.* 2017) and expands its analysis.

Two more case studies can be found in the Supplementary Text with Supplementary Figs S14 and S15 as well as Supplementary Tables S10 and S11. They are both re-analysis of Hemagglutinin data from Influenza A from prior publications (Chang *et al.* 2020, 2022). The first case shows the ability of RAMZIS to identify global differences based off sample group exclusive observations. The second case shows RAMZIS ability to identify overall shifts in glycosylation quantification patterns and its utility in informing experimental design.

### 3.1 AGP case study premise

A prior experiment found that as sample heterogeneity increases, the ability to identify and quantify glycopeptides decreases (Khatri *et al.* 2017). This problem can be alleviated using an informed search space, one that uses proteomic and glycomic data to create a more targeted search space. These informed search spaces increase the confidence of our results and the likelihood of identifying and quantifying glycopeptides. The prior experiment looked at purified alpha-1-acid glycoprotein (AGP; Uniprot: P02763) (The UniProt Consortium 2023), as well as AGP spiked into increasing amounts of human serum glycoproteins.

These data were analyzed using a standard GlycReSoft analysis workflow using default settings (Chang *et al*. 2022). Peaks Studio 8 (Zhang *et al*. 2012) was used for the proteomics search. GlycReSoft was used for the glycan search. Both the glycomics and proteomics databases were used to generate the informed search spaces. The naive search spaces that were used to compare against the informed search space were combinatorial glycan search spaces attached to all proteins expected to be in the mixture. All searches were performed at 5% FDR and used fixed carbamidomethylation and variable oxidation, deamidation, and pyro-glutamination.

#### 3.1.1 AGP: naive versus informed in a homogeneous mixture

In glycoproteomics studies, it is tempting to make assumptions about the glycoprotein purity. We showed that it is better to build a glycoproteomics search space based on measured glycome and proteome information (Khatri *et al.* 2017). A naïve search space uses sets of glycan compositions from public databases and assumes that the target glycoprotein is pure. An informed search space uses measured glycomes and proteomes to define the set of theoretical glycopeptides used to search the data.

**Figure 1.**
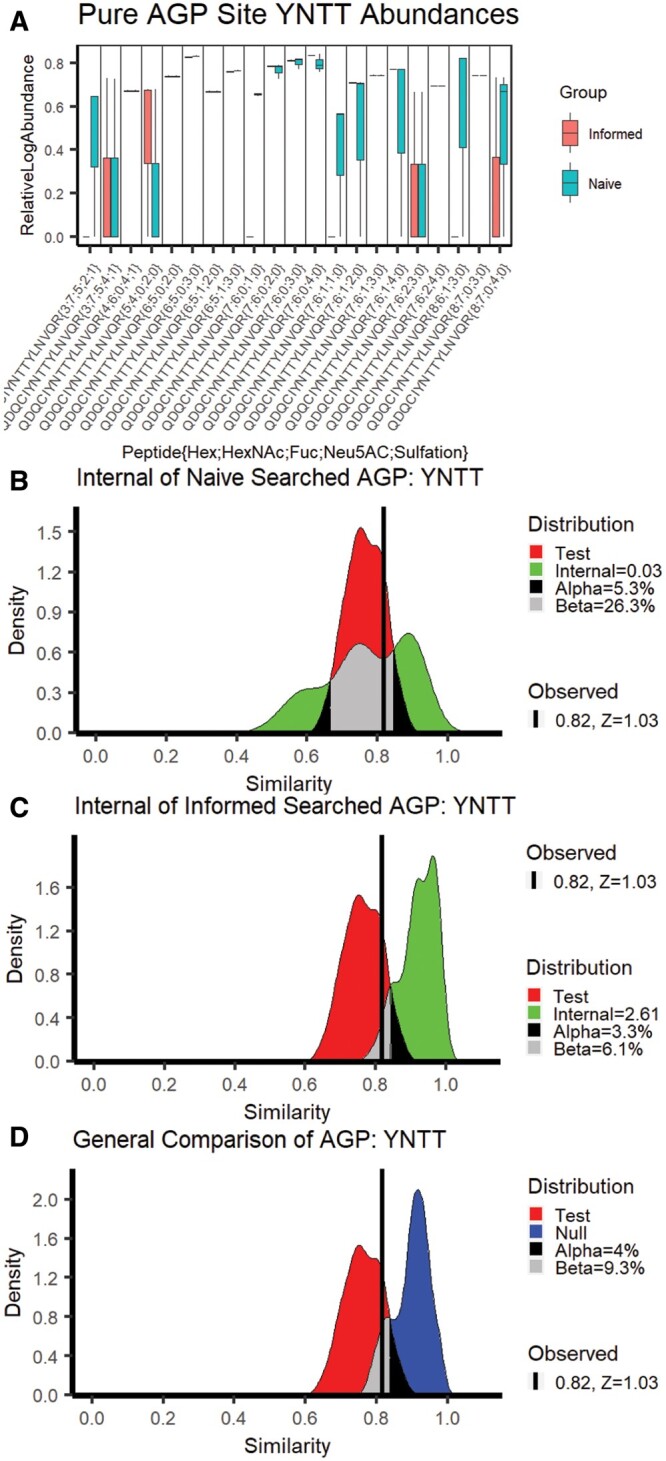
AGP Naïve versus Informed Search Space Comparison. (A) The relative log abundances of the glycopeptides observed in pure AGP samples; the Informed search values are in red, and the Naïve search values are in teal; these have been standardized by TIC. (B) The Internal Similarity of the Naive search space with IC = 0.03, FPR = 5.3%, FNR = 26.3%. (C) The Internal Similarity of the Naive search results with IC = 2.61, FPR = 3.3%, FNR = 6.1%. (D) The general comparison between the Test and Null similarities of the Naive and Informed search space results with FNR = 4%, FPR = 9.3%, and has the observed similarity at 0.82 with a Test Similarity *z*-score of 1.03.

We applied RAMZIS to the published data from that study and found that, even in a low complexity mixture of purified AGP, we identified differences in the overall glycosylation pattern between naïve and informed search spaces as shown in Fig. 1. This analysis focuses on the fourth AGP glycosite located at the 93rd amino acid, herein YNTT. RAMZIS identified an outlier sample from this dataset, reducing the sample size to three separate LC runs; this outlier identification and removal process can be found in Supplementary Fig. S11. The remaining multimodality is due to a higher quality replicate type outlier.

In Fig. 1A, the standardized log abundances of the glycopeptides are displayed in a pairwise boxplot, with the Informed search results in red on the left and the Naïve in teal on the right. The quantifications appear highly consistent with notable visual exceptions. Boxplots, such as the abundances at YNNT{3;7;5;4;1}, indicate that two of three samples are zero, with the third at the line end point.

Figure 1B and C is both Internal similarity plots with the Test similarity in red and the Internal similarities in green. Figure 1B shows the Naïve search’s Internal Similarity with an IC of 0.03, an FPR of 5.3%, and an FNR of 26.3%. These fail all quality metrics, which indicates that the Naïve has insufficient quality for a comparison against the Informed. Figure 1C shows the Internal similarity of the Informed search with an IC of 2.61, an FPR of 3.3%, and an FNR of 6.1%. These pass all quality metrics, indicating that the Informed has a high enough data quality for this comparison. The Test similarity meets its own quality threshold with the observed similarity of the original comparison at 0.82 with a *z*-score of 1.03, <3.

The general similarity comparison is shown in Fig. 1D, comparing the Test to the Null similarity distributions. The Null is in blue, and there is a 4% FPR and a 9.3% FNR. Without the data quality problems seen in Naïve, the glycosylation patterns could be considered differentiable, but the comparison cannot be made reliably. The glycopeptides that are ranked to be the most likely contributors to differentiation are shown in Table 1 with their quality assessment and ranking value; the glycopeptides are listed in ranked order. None of these glycopeptides have contribution *z*-scores <−2, and only one passes quality assurance metrics. Missing value-induced quality issues are the primary contributors to many of the differences seen here; four of the top five ranked glycopeptides had glycans not present in the informed search space, and one {Hex 3;HexNAc 7;Fuc 5;NeuAc 2;Sulfate 1} is not biosynthetically likely, as it is over-fucosylated and has too few hexose for a standard lactosamine extension (Stanley *et al.* 2022), and was unobserved in the glycomic data.

**Table 1.** Glycopeptide rankings in Naïve versus Informed AGP: YNTT.^a^

Glycopeptide	*z*-Score	Quality	Likely failure cause
YnNT{7;6;0;1;0}	−1.947	F	Unseen in file 2
YnNT{6;5;0;3;0}	−0.934	T	Passed
YnNT{7;6;1;1;0}	−0.886	F	Low quality in both
YnNT{3;7;5;2;1}	−0.884	F	Low quality in both
YnNT{8;6;1;3;0}	−0.880	F	Low quality in both

a A truncated, reformatted ranking data summary from RAMZIS. Each row shows a different glycopeptide’s identity (PEP{Hex;HexNAc;Fuc;Neu5Ac;Sulfate}), contribution *z*-score, quality indicator, and probable quality failure cause.

#### 3.1.2 AGP: comparing across heterogeneities

**Figure 2.**
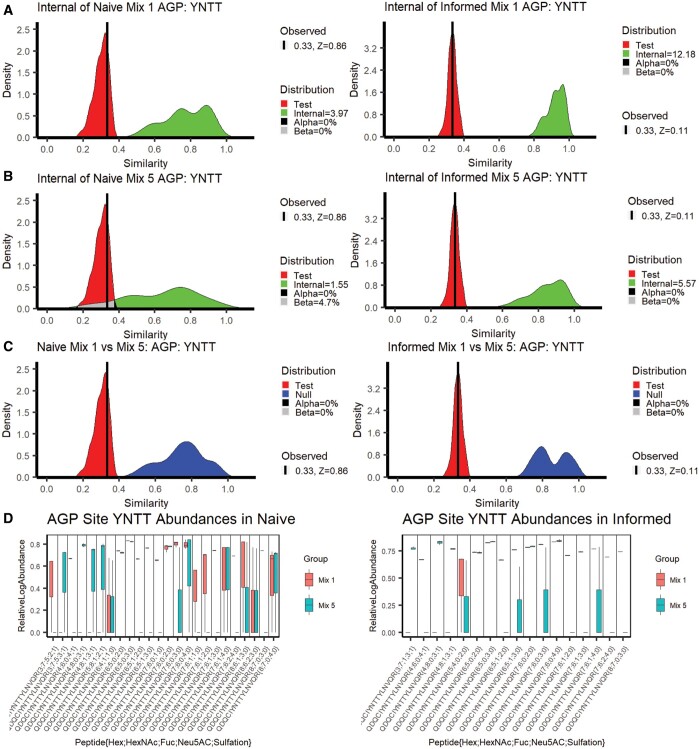
Related similarity comparisons in AGP mixtures. A side-by-side display of two related similarity comparisons: purified AGP compared to AGP in a heterogeneous mixture. The left column is the naïve search while the right is the informed. (A) Internal similarities of pure AGP. Naive AGP IC = 3.97, 0% FPR & FNR; Informed AGP: IC = 12.18, 0% FPR & FNR. (B) Internal similarities of Mix 5. Naive AGP: IC = 1.55, 0% FPR, 4.7% FNR; Informed AGP: IC = 5.57, 0% FPR & FNR. (C) Comparisons of Test and Null distributions. The Naive comparison had a 0% FPR and a 0% FNR; the Informed comparison had a 0% FPR and a 0% FNR. RAMZIS simulated the comparison sufficiently for both identification methods with Test *z*-scores of 0.86 and 0.11 for the Naïve and the Informed, respectively. (D) The relative log abundances used to calculate the above similarities are displayed by glycopeptide in a box plot; Mix 1 is darker and on the left of each pair while Mix 5 is lighter and on the right of each pair. These have been standardized by TIC and scaled according to a linear model by 1.067 and 1.084 for Naïve and Informed, respectively. Mix 1 Naïve has a 70% presence rate; Mix 5 Naïve has a 39% presence rate; Mix 1 Informed has an 80% presence rate, and Informed has a 66% presence rate.

RAMZIS allows for deeper understanding of the differences induced by search space differences and complexity increases. To assess the impact of complexity, we present the results of two comparisons between purified AGP (Mix 1) and Mix 5, a sample of equal parts AGP, transferrin, fetuin, haptoglobin, and alpha-2-macroglobin. The left-hand side of Fig. 2 pertains to a Naively searched analysis while the right-hand side pertains to an Informed search analysis; all graphs in Fig. 2 are in relation to AGP glycosite YNNT. Figure 2A shows the Internal similarity distributions of Mix 1 in the comparison of Mix 1 and 5. The left of Fig. 2A shows the Naïve Mix 1 to be completely disjoint with the Test similarity, leading to an IC of 3.97, an FPR and an FNR of 0%. The Informed search on the right of Fig. 2A is also disjoint with an IC of 12.18 and 0% FPR and FNR. Both Mix 1 Internal similarities pass all quality metrics, and the Test Similarity of both is simulated well by RAMZIS with observed similarities with *z*-scores less of 0.86 and 0.11 for Naïve and Informed, respectively.

Figure 2B shows the Internal Similarity distributions of Mix 5 shows divergence in behaviors between Naïve and Informed searches. The Naïve Mix 5 on the left has an IC of 1.55, an FPR of 0%, and an FNR of 4.7%, while the Informed Mix 5 has an IC of 5.57 and an FPR and FNR of 0%. The Informed gives high enough data quality to reliably compare the two datasets, while the Naïve search space fails at the higher complexity. Subtle multimodality is present, but Mix 5 had an *n* = 3, so outlier removal was not performed. The General Comparisons in Fig. 2C both show completely disjoint Null and Test distributions for both the Naïve and Informed comparisons with 0% FNR and FPR for both. Figure 2D shows the boxplots of the TIC standardized abundances. In the Naïve, Mix 1 has 4 absent glycopeptides while Mix 5 has 10; in the Informed, Mix 1 has three absent glycopeptides while Mix 5 has six. Multiplicative scaling terms were applied to both Mix 5 abundance datasets to account for a semi-uniform shift of abundances that were not reflected by the TIC standardizations. This rescaling does not substantially change the overall comparisons, and a version of Fig. 2 without this shift can be found in the Supplementary Material. Table 2 shows the ranking information for the highest ranked glycopeptides in the Naïve comparison of Mix 1 and Mix 5. All of these glycopeptides are absent from Mix 5 and have significant contributions to dissimilarity. One of the glycopeptides {Hex 4;HexNAc 6;NeuAc 4;Sulfate 1} is biosynthetically unlikely, as the high sialylation relative to the amount of hexose makes the normal lactosamine extension impossible (Stanley *et al.* 2022), but it was identified in the glycomic data. Examination of the GlycReSoft files shows that its identifying spectra share information with more biosynthetically likely glycans, but that in some cases, it was selected based off of MS1 error. A full Naïve comparison ranking summary table is available in Supplementary Table S6.

**Table 2.** GP ranking of Naïve Mix 1 versus Mix 5: AGP: YNTT.^a^

Glycopeptide	*z*-Score	Quality	Likely failure cause
YnTT{7;6;0;1;0}	−2.017	F	UnseenIn_File2
YnTT{6;5;1;2;0}	−2.015	F	UnseenIn_File2
YnTT{4;6;0;4;1}	−2.015	F	UnseenIn_File2
YnTT{7;6;2;3;0}	−2.008	F	UnseenIn_File2
YnTT{7;6;1;3;0}	−2.000	F	UnseenIn_File2

a A truncated, reformatted ranking data summary from RAMZIS. Each row shows a different glycopeptide’s identity (PEP{Hex;HexNAc;Fuc;Neu5Ac;Sulfate}), contribution *z*-score, quality indicator, and probable quality failure cause.

Table 3 shows the top five ranked glycopeptides of the Informed Mix 1 to Mix 5 comparison. All five are unseen in Mix 5, and are all significant contributors to dissimilarity. The same unlikely glycopeptide from the Naive is seen again. A full Informed comparison ranking summary table is available in Supplementary Table S8. An unscaled analysis can be found in Supplementary Figs S12 and S13 with their ranking summaries, Supplementary Tables S7 and S9 for Naïve and Informed, respectively.

**Table 3.** GP ranking of Informed Mix 1 versus Mix 5: AGP: YNTT.^a^

Glycopeptide	*z*-Score	Quality	Likely failure cause
YnTT{6;5;1;2;0}	−2.017	F	UnseenIn_File2
YnTT{4;6;0;4;1}	−2.016	F	UnseenIn_File2
YnTT{7;6;1;2;0}	−2.011	F	UnseenIn_File2
YnTT{7;6;2;4;0}	−2.008	F	UnseenIn_File2
YnTT{7;6;1;3;0}	−2.001	F	UnseenIn_File2

a A truncated, reformatted ranking data summary from RAMZIS. Each row shows a different glycopeptide’s identity (PEP{Hex;HexNAc;Fuc;Neu5Ac;Sulfate}), contribution *z*-score, quality indicator, and probable quality failure cause.

## 4 Discussion

### 4.1 AGP results interpretation

The study that produced these data showed that the use of an informed search space improved glycopeptide identification confidence. RAMZIS has confirmed and helped quantify this phenomenon.

There is an observable difference between Naïve and Informed search analysis even when just looking at purified AGP. The ranking information quantitatively confirms the visually apparent fact that this difference is not from differences in quantification, but instead from missingness caused by an overly broad naïve search space. This analysis enables a more stringent confirmation than traditional statistics allows for.

This case-study also confirms the decrease in identification confidence that comes with increased sample complexity. The Informed comparison between Mix 1 and Mix 5 shows differentiability between the two that is primarily caused by missingness in Mix 5, despite Mix 5 actually having larger glycan and protein search spaces. As mixture complexity increases, the likelihood of identifying lower abundance glycopeptides decreases. While confounded by the non-uniform scaling, there is still evidence that quantification is susceptible to this same loss in confidence. The glycopeptides that passed quality assurance metrics were still contributors to difference and were more so prior to the artificial adjustment made for the purpose of a more conservative analysis. This change in quantification confidence is not unreasonable as with less confidence in an identification there may become fewer points in the elution from which to find the AUC.

The Naïve comparison of Mix 1 and Mix 5 shows the two issues compounding upon one another. The naively searched Mix 5 has a much higher rate of missingness, but instead of forcing differentiation from Mix 1, it appears as a low-quality subset of it. We can identify outlier issues via modality in Mix 5, and while the high degree of overlap in the general comparison should be expected of a glycosite with the same glycosylation, the degree of overlap between the Internal and Test similarities of Mix 5 show a lower degree of internal consistency than of Mix 1. There are small yet observable differences in quantification even after rescaling, and the quantifications themselves are more variable in the naïve search space than the informed as seen in Fig. 2D.

One of the most important problems in glycoproteomics is determination of assignment confidence for a complex sample. We have demonstrated the use of RAMZIS to compare glycopeptide assignments and quantifications between low and high complexity glycoprotein mixtures. We found that the difference between informed and naïve searches are discernable even at low complexity, and that the effects become magnified as complexity increases. As mixture complexity increases, the identifications with higher signal values become the only ones identified, and they become less consistently quantified, especially in uninformed search spaces.

### 4.2 RAMZIS broader impacts

As most of the questions that glycoproteomics seeks to answer are in inherently higher complexity systems, the ability to assess data quality as part of the comparative process is key. The ability to understand not only that there are differences, but to be able to quantitatively determine the source of these differences, will be necessary for a fuller understanding in both improving our methods and interpreting our experiments.

Our data quality decreases with every additional glycopeptide that must be tracked, and we fight against technological limitations of throughput in our attempts to quantify the entire glycoproteome. We must know when our attempts to surpass these throughput problems become overwhelmingly deleterious to our ability to reliably quantify, and we must be able to identify how exactly they are so; this knowledge will enable us to fine tune methods, determine acceptable losses, and ideally, perfect them to work around those deleterious effects.

RAMZIS is an effective tool for modeling pairwise N-glycopeptide comparisons and gives a clear readout of assignment confidence that will guide users in appropriate interpretation of their glycoproteomics data. It is a step in the road to developing better methods and performing more consistent analysis of glycoproteomics. With it users will be able to identify outliers, assess data consistency, perform heuristic pairwise glycosite comparisons, and target potential identifications that are the sources of error or differentiation.

The existing framework of the tool is set-up for future expansions. As it currently stands, the package is relatively computationally intensive, making thousands of matrix comparisons. While these comparisons are core to the actual operation of the package, there is likely room for improvement in computational resource expenditure, and the package would benefit greatly from in built parallelization.

Future expansion of the application is more open ended. Currently, we are testing the viability of incorporating information from the biosynthetic network into RAMZIS, which would enable a better understanding of the rankings and help to identify patterns if any glycopeptides are behaving differently than their related network neighbors. It is also our hope that use of the biosynthetic network may be used to similarly lessen the impact of missing values by identifying the above aberrant gradient behaviors in conjunction with missing values.

RAMZIS is ultimately a quality assurance tool, and it should be backed up by other statistical tests on likely candidates with more targeted data; it is a guide, not a determinant, and allows users to answer the question: “Am I able to ask the question I want to ask?” If the Internal Similarity distributions are sufficiently well defined, then it is possible for the user to ask the next question: “Are these glycosites likely to be differentiable?” And while it does enable differentiating glycosylation patterns at a glycosite, the rankings it provides are meant to steer the user toward the likely answers will inform further experimental investigations to help answer the final question: “How are these glycosites different?”

## Supplementary Material

vbae012_Supplementary_Data

## Data Availability

The data underlying this article will be shared on reasonable request to the corresponding author.
