# Supplementary material for: RAMZIS: a bioinformatic toolkit for rigorous assessment of the alterations to glycoprotein composition that occur during biological processes
Source: Bioinform Adv. 2024 Jan 25;4(1):vbae012. doi: 10.1093/bioadv/vbae012 (PMC10879752; doi:10.1093/bioadv/vbae012)
Supplement: vbae012_Supplementary_Data [file vbae012_supplementary_data.pdf]

RAMZIS Supplemental

Authors: WE Hackett, DK Chang, L Carvalho, J Zaia

This document contains elaboration on the workings of RAMZIS and presents more of the testing done.

Supplemental Methods... 2
Algorithm Workflow...2
Supplemental Figure 1: Algorithm Workflow...2
Input Data...3
Data Format Example...3
Supplemental Table 1...3
Standardization & Relativization...3
Data Simulation via Bootstrap...5
Similarity Comparison...5
False Positive and False Negative Rates...6
Internal Confidence Score...6
Ranking Identifications...7
Quality Metrics...7
Ranking by Contribution...7
Theoretical Data Generation...8
Outlier Detection...9
Supplemental Results... 10
Theoretical Examples...10
Supplemental Figure 2: Theoretical Data Generation...10
Theoretical Case: Same Underlying Distribution...11
Supplemental Figure 3: Theoretical: Same Underlying...11
Supplemental Figure 4: Theoretical: Same Underlying Increased Variance.............12
Supplemental Figure 5: Theoretical: Group A vs Itself...13
Supplemental Table 2: Theoretical Rankings of Same + Inc...13
Theoretical Case: Completely Different...14
Supplemental Table 3: Theoretical Rankings of Completely Different.....................15
Supplemental Figure 6: Theoretical: Completely Different.....................................16
Theoretical Case: Major vs Minor Deviations...16
Major Deviation...16
Supplemental Figure 7: Theoretical: Major Deviation...17
Supplemental Figure 8: Theoretical: Major Deviation in Expected Variance............18
Supplemental Figure 9: Minor Deviations: Overall changes without Individual significance...19
Supplemental Table 4: Ranking Minor Deviations...20
Theoretical Case: Outlier Identification...20

Supplemental Figure 10: Theoretical: Outlier Removal.......... 20
AGP Case-Study: Pure AGP.......... 22
Outlier Removal.......... 22
Supplemental Figure 11: AGP: Outlier Removal..........23
Full Ranking Summary..........24
Supplemental Table 5: Pure AGP Glycopeptide Rankings.......... 24
AGP Case-Study: Mix 1 vs Mix 5..........25
Naive Scaled..........25
Full Ranking Summary..........25
Supplemental Table 6: Naive: Mix 1 vs Mix 5 Scaled.......... 25
Naive Unscaled..........26
Similarity Analysis.......... 26
Supplemental Figure 12: AGP: Naive Unscaled: Mix 1 vs Mix 5.......... 26
Full Ranking Summary..........27
Informed..........28
Full Ranking Summary..........28
Supplemental Table 8: Informed: Mix 1 vs Mix 5 Scaled.......... 28
Informed Unscaled..........28
Similarity Analysis.......... 28
Supplemental Figure 13: AGP: Informed Unscaled: Mix 1 vs Mix 5.......... 29
Full Ranking Summary..........29
Supplemental Table 9: Informed: Mix 1 vs Mix 5 Unscaled.......... 29
Biological True Positive Case Studies from Prior Works.......... 30
Phil82: Galactosidase's Impact on Hemagglutinin..........30
Supplemental Table 10: Phil82: HA Native vs HA Gal..........31
Supplemental Figure 14: Phil 82 HA at 483-NGTY: Native vs Galactosidase.......... 32
Supplemental Table 10: Rankings of Phil 82 HA at 483-NGTY: Native vs Gal..........33
SWZ13: Glycosylation Differences of HA Between Chicken and Dog..........34
Supplemental Figure 15: MDCK vs Egg in HA at site 165-NVTM.......... 35
Supplemental Table 11: Ranking Summary of MDCK vs Egg at site 165-NVTM...... 36

Supplemental Methods

Algorithm Workflow

Supplemental Figure 1: Algorithm Workflow

Figure 1. Algorithm Workflow

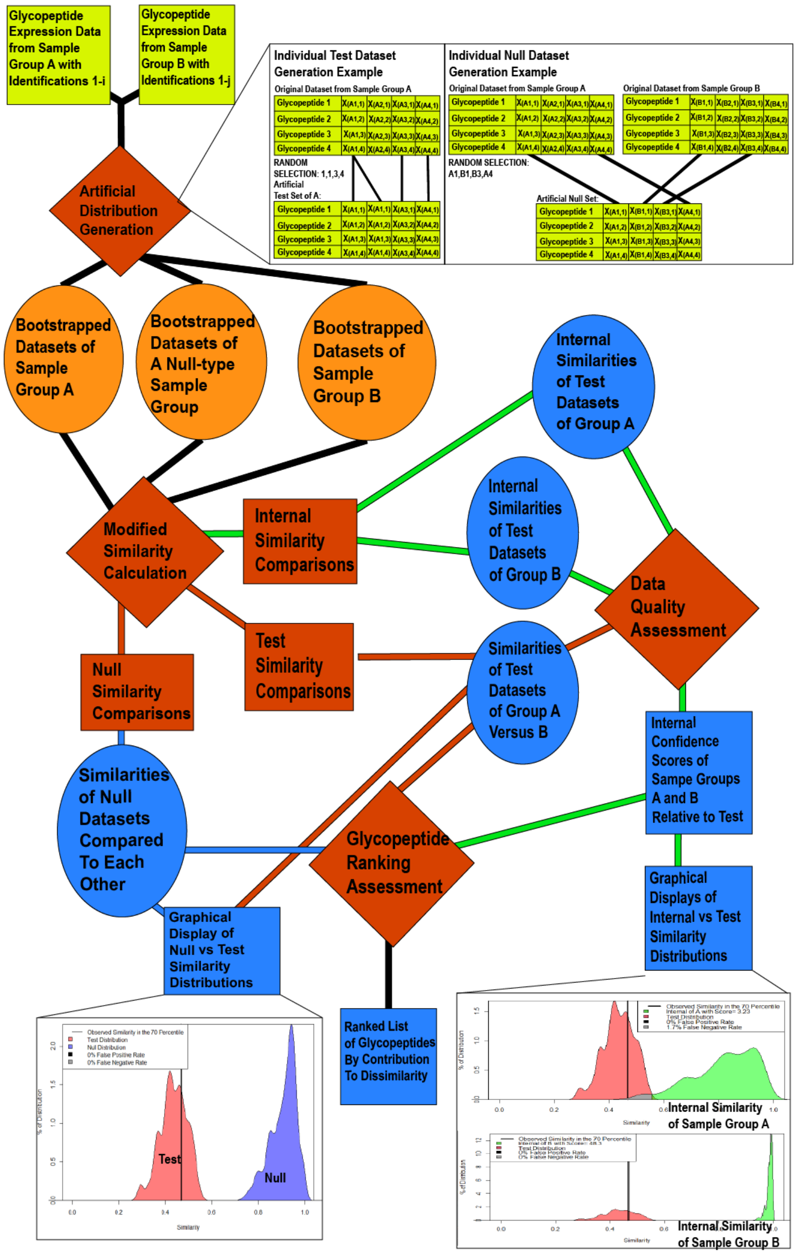

Supplemental Figure 1 depicts the algorithmic workflow of RAMZIS. Inputs are depicted in yellow; processes are depicted in red; temporary data are in orange; outputs are in blue. Circles are datasets; squares are user interpretable.

- The user provides two glycopeptide expression datasets, herein called Group A and Group B
- Those datasets undergo bootstrap sampling to produce 3 different kinds of simulated datasets: Samplings of Group A, Samplings of Group B, Samplings of Groups A & B at the same time.
- These datasets are the subjected to different kinds of similarity comparisons. The Samplings of A produce Internal Similarities of Group A. The Samplings of B produce Internal Similarities of Group B. The Samplings of A compared to B produce Test Similarities. The Joint samplings produce Null similarities.
- These similarities are fed into data quality assessments and glycopeptide ranking assessments.
- The similarities and their data assessments are used to produce comparative similarity graphs.

Input Data

RAMZIS can be used to format data from GlycReSoft outputs, but if you are using a different tool for Glycopeptide identification and quantification, you will need to do your own formatting. It's also recommended to input a normalization vector.

Data Format Example

The universal data format that RAMZIS accepts is a matrix with glycopeptides for rows and samples for columns. They're expected to have row and column names. Row names are how RAMZIS knows which identifications are the same between files. The cells are filled with The matrix of one file looks something like this:

Supplemental Table 1

	Sample_1	SampleB	PatientIII	NumberD
GlyPep1	1230000	1320000	1290000	120000
GlyPep2	95000	105000	100000	90000
GlyPep3	20000	25000	22000	18000

RAMZIS requires two of these files as input. It will also accept dataframes formatted in this fashion.

The names of the samples (columns) don't matter so long as they don't break R formatting rules. Glycopeptides can similarly be named anything so long as they are consistent between files. It will not combine missed cleavages into a single glycosite, and any such data manipulations should be done prior to input into RAMZIS. RAMZIS can be used to compare PTMs other than glycosylation, and if other PTMs are included the glycopeptide names, they will be treated as fully separate glycopeptides from equivalent glycopeptides without those PTMs as RAMZIS uses an exact match system.

Standardization & Relativization

There are multiple standardization methods involved in the initial data processing.

Log Transformation

Users may choose to not have their data undergo log transformation(logopt=F), but it is recommended(Default: logopt=T). RAMZIS uses the default log() function of R, which is the natural log. Log transformation of most glycopeptide abundance data scales the overall data such that it becomes more normally distributed.

Minimum Observation Requirements

For a glycopeptide to be considered real it must be seen twice(Default: kmin=2) between the two datasets. This requirement for cross dataset minimums is based on the idea that the two datasets, until proven otherwise, are from the same underlying glycosylation distribution. The number of observations required to be seen may be changed, and the cross dataset minimum may be disabled. Internal Similarity is calculated without a minimum observation requirement to make comparisons more conservative; low observation identifications reduce the overall

similarity and make overlap between the Test and Internal more likely. Users may also set the internal minimum observation threshold. (Default: kmin_int=1)

Normalization aka Standardization
Data is not normalized in RAMZIS, but it can be standardized. Normalization, the process of turning values into z-scores, is avoided as the similarity metric requires positive values. Standardization, the process of scaling abundance values between samples and datasets, is optional but recommended. (Default: normvec=list("None","None")

RAMZIS expects two vectors in a list for its standardization process, wherein the first vector should correspond in length to the number of samples in the first input dataset and the second vector should correspond in length to the number of samples in the second input dataset. The first sample in the first dataset will have all its glycopeptides multiplied by the first value of the first vector in the list, the second sample in the first dataset will be multiplied by the second value of the first vector and so on.

The source of these standardization factors is up to the user, but it is recommended to use a factor derived from the TIC- the Total Ion Chromatogram, a single summation of the entire signal produced by the elution. If using the default log transformation, take the log of each sample's TIC and then take the inverse of it. (scaling_factor[1]=1/log(TIC[1]) If not using the log transform then simply take the inverse of the TIC. (scaling_factor_nolog[1]=1/TIC[1])

Another potential source of standardization is from protein abundance data. If a protein is more abundant in one sample group over another, then the TIC may not properly adjust. In this case, the use of a more complex scaling term would be appropriate. This could take the form of a series of conversion factors like this:

$$ScalingFactor(Sample_i, Protein_j) = \frac{1}{ln(TIC_i)} * \frac{E[Abundance_j]}{Abundance_{j,i}}$$

Where i is a sample index, and j is a given protein. E[Abundance(j)] would be the mean abundance of the protein in all samples across datasets. Abundance(j,i) would be the abundance of the protein j in the proteomics data that corresponds to sample i.

Joint Relativization
Joint Relativization is performed when no standardization vectors are given; it can be enabled to occur even then (Default: rel_force=F). Relativization divides the abundances given by It defaults to making all abundances relative to the largest summed abundance of any sample in both datasets. (Default: rel="Joint") This default assumes that the overall signal is equivalent between files, and that differences between the files can be believed to be from concentration rather than signal response variance. There are also options to make abundances relative to the largest summed abundance within a dataset (rel="Within"), to be left as is (rel="AsIs"), to have each sample relative to its own total abundance (rel="Self"), or have all values relative to a specified value (rel=NUMERIC).

Data Simulation via Bootstrap

Test Dataset Generation
Each input dataset will have simulated data generated from it. A simulated dataset will be a random sampling by column with replacement. 100 samplings are produced, and in lower sample number projects the samplings will be combinatorially exhaustive, but will not contain repeats and thus have fewer than 100 samplings. These Test datasets will be used to produce the Internal Similarity Distributions, by comparing all those of a given origin dataset to all others in the origin dataset, and the Test Similarity Distribution, by comparing all samplings of one dataset to all samplings of the other dataset.

Null Dataset Generation
To generate the two Null Datasets required, random samplings of a joint dataset are taken with probability equally split between sample groups, and probability split within those groups equally to each sample. There is a minimum number of samples required from each dataset so that there are no full exclusions in a given sampling. Repeated samplings are not allowed. If the origin datasets have equivalent sample size then the samplings will be produced together and then split evenly to produce two Null samplings. If the origin datasets have unequal sample sizes then they are produced separately.

Similarity Comparison

The similarity equation is largely explained in the article itself. This section is to denote two features that can be modified within the analysis.

Missing Value Correction
This is not an imputation option. As a default, RAMZIS fills in unseen glycopeptides with zeros. (Default MVCorrection=T) If this option is disabled (MVCorrection=F) then the P terms of the similarity equation are disabled, meaning that missingness will no longer factor into similarity. This substantially changes similarity comparisons with high degrees of missingness and may induce errors in cases of complete absence.

Distance Scaling
Distance scaling (K) can be changed. (Default: mn=F) As a default, it has K=1+mean(Presence(A),Presence(B)) such that K can be at most 2, meaning that in cases of maximal distance the numerator will be halved. If this option is given a numeric value, it will use that value. This is convenient if the user wishes to increase the impact of the manhattan distance between glycopeptide abundances.

False Positive and False Negative Rates

Equations 3 and 4 are presented in the paper are simplified with T as the density of the Test Distribution; R as the density of a Reference distribution; x as the point at which Test has a lower density than the Reference. These densities are approximated by the empirical cumulative distribution as determined by R's ecdf() function.

$$AlphaOverlap(T,R) = \frac{\int_x^1 T}{T+R-(\int_x^1 T+\int_0^x R)} \quad (3)$$

$$BetaOverlap(T,R) = \frac{\int_0^x R}{T+R-(\int_x^1 T+\int_0^x R)} \quad (4)$$

Are simplified forms as there can be multiple inflection points wherein the reference and test distributions cross over one another. Inflection points are determined using R's stats::density() function with set bounds from -0.1 to 1.1; once generated these densities are scaled to their totals. More accurate versions of equations 3 and 4 can be represented here:

$$AlphaOverlap(T,R) = \frac{\sum_i^{(n)}(ecdf(T)[i[2]]-ecdf(T)[i[1]])}{T+R-(\sum_i^{(n)}(ecdf(T)[i[2]]-ecdf(T)[i[1]])+\sum_j^{(m)}(ecdf(R)[j[2]]-ecdf(R)[j[1]]))} \quad (3)$$

$$BetaOverlap(T,R) = \frac{\sum_j^{(m)}(ecdf(R)[j[2]]-ecdf(R)[j[1]])}{T+R-(\sum_i^{(n)}(ecdf(T)[i[2]]-ecdf(T)[i[2]])+\sum_j^{(m)}(ecdf(R)[j[2]]-ecdf(R)[j[1]]))} \quad (4)$$

Here, n represents pairs of similarities, i, where i[1] is where the Test density becomes lower than the Reference and i[2] is the last similarity that the Test is lower than the Reference; m represents pairs of similarities, j, where j[1] is where the Reference density becomes lower than the Test and j[2] is the last similarity where the Reference is lower than the Test. No region is double counted.

Internal Confidence Score

Weighted Z-Score

The current Internal Confidence Score follows equation 5:

$$InternalScore = \left(\frac{\mu_{Test}-\mu_{Internal}}{\sigma_{Internal}}\right) * 10^{-(\alpha+\beta)}$$

Where μ are means of their respective similarity distributions; σ is the standard deviation; α is the false positive rate from equation 3, and □ is the false negative rate from equation 4. This weighted z-score is penalized by larger overlaps to avoid overconfidence in case of long tailed distributions, which are more common in non-log transformed similarities. Joint overlaps greater than the combined 0.25 (from 0.05 and 0.20 for FPR and FNR respectively) will more than halve the z-score.

Prior Version Scoring: Pseudo-Kurtosis

A prior version of the Confidence score used the similarity density height to variance as a form of kurtosis like measurement. That used a threshold of Score>100, meaning that an Internal Similarity Distribution would have a density plot height 100 times greater than the similarity variance. This was heavily reliant on density plotting, not necessarily user interpretable, and not as well grounded in mathematics.

Ranking Identifications

The ranking and quality assessment of individual glycopeptides contributions to similarity rely heavily on two different related measurements. That is the unweighted contribution to similarity, and the weighted contribution.

$$UnweightedContribution(g) = A_g B_g K^{-d(A_g, B_g)}$$

$$WeightedContribution(g) = \frac{A_g B_g K^{-d(A_g, B_g)}}{\sum_i^G (A_i^2) + \sum_i^G (B_i^2) - \sum_i^G (A_i B_i K^{-d(A_i, B_i)})}$$

The unweighted contribution is just the numerator of the modified similarity equation while the weighted contribution is adjusted by the denominator term from the similarity equation. The former is used in ranking, while the latter is used in quality assessment.

Quality Metrics

As the contributions have much smaller distributions, quality thresholds for them are joined from an alpha of 0.05 and beta of 0.20 to a joint overlap (of Internal and Test) proportion of 0.25. The Internal Similarity contribution should be greater than the Test if using the Weighted Contributions. Other quality metrics have been identified, but are not currently implemented into the main workflow as they are still being tested.

Weighted Contributions

The weighted contributions are used for quality assessment as they provide a more conservative measure of overlap between the Internal and Test distributions. If a user desires for it to be consistent with the ranking process, they may specify to the less conservative unweighted contributions. (QualityInfo=”Numerator”)

Ranking by Contribution

The ranking of an individual glycopeptide uses the average z-score of its Test similarity contribution in relation to the Null Similarity contribution distribution.

$$RankingInfo = \frac{1}{n}\sum_i^n \left(\frac{Contribution_{Test,i} - \mu_{Contribution,Null}}{\sigma_{Contribution,Null}}\right)$$

Where i is one comparison of all n Test similarity comparisons. The lower the z-score, the more likely it is that the glycopeptide in question contributes strongly to the differences in the overall similarity distribution.

Unweighted Contribution

While the unweighted contributions are less conservative when used in the quality assessment step, the weighted contributions are misleading when used as the ranking information. Missing

values are the cause of this phenomenon. When there is a complete absence of a glycopeptide in one dataset, then the Test contributions for that glycopeptide will all be zero, but the Null similarity contributions will have high variance, and may include zeros. This produces a high variability in the Null, leading to a lower absolute z-score for that glycopeptide, which isn't problematic, highly variant glycopeptides should not be considered consistent contributors to dissimilarity. But other glycopeptides will have higher weighted contributions if there are low Null values boosting their value.

Use of weighted contributions for ranking information causes missing values to be devalued in rankings. Weighted contributions are biased by the effect of missing value glycopeptides on fully present glycopeptides, and so using weighted contributions can lead to spurious conclusions. Unweighted contribution based ranking, while less conservative, acknowledges the impact of missing values and does not boost other glycopeptides.

Should a user wish to use weighted contributions they may do so, but it is not recommended. (RankingInfo="WeightedContributions")

Alternative Quality Metric When Using Weighted Contributions

If using the weighted contributions for ranking, a secondary confirmation on a significant glycopeptide ranking can be helpful to be sure that there is no bias from missing values, however this is not a guaranteed filter. This second confirmation is a comparison of the normalized contribution of the Test against the normalized contribution of the Null. For a given glycopeptide, generate the z-score of the contribution compared to all other contributions in the similarity distribution in a leave-one out fashion.

$$Z(g_{SimDis}|G_{SimDis}) = \frac{g_{SimDis} - \mu_{G!=g}}{\sigma_{G!=g}}$$

Where g_{SimDis} is the similarity contribution of a given glycopeptide in a Similarity Distribution SimDis; G_{SimDis} are all contributions of all glycopeptides in SimDis; $\mu_{G!=g}$ is the mean of all contributions in G excluding the given glycopeptide g, and $\sigma_{G!=g}$ is the standard deviation of all contributions in G excluding the given glycopeptide g.

This z-score is generated for the Test and the Null for each glycopeptide, and only glycopeptides that have Test z-scores lower than their Null z-scores can be considered as a potentially significant contributor to dissimilarity.

Theoretical Data Generation

The theoretical data generator is good for testing potential behaviors in RAMZIS. It allows a user to produce RAMZIS formatted datasets to their specifications. The user controls the numbers of samples and glycopeptides, as well as their variability, missingness, and their means.

Data Generation via Beta Distribution Sampling

The data points are sampled from a beta distribution. Beta distributions have means defined by the ratio of an alpha parameter to a beta parameter. By inputting a list of alphas and betas equal to the number of glycopeptides, the user can define the mean for each theoretical glycopeptide. Variance of a beta distribution is proportional to the size of the alpha and beta, the greater they are the less variant they are. Use of this property allows users to specify how consistent their data is separately from their alpha and beta inputs, and it can even be controlled on a per glycopeptide level. The data generation uses three variables to control the variance: 1) the number of significant figures kept of the initial mean samplings. (Default=2) This number is turned into a ratio of alpha and beta to replicate the mean in sampling; the more numbers kept after the decimal, the larger the numbers in the ratio, the smaller the variance. 2) A ratio multiplier (Default=10) which scales the numbers in the ratio, and 3) a multiplier exponent (Default=1) to more quickly change the variance by exponentiating the multiplier.

Missingness Generation

After data generation via beta distribution, missingness is introduced to the dataset. Based off of an average presence input by the user, each glycopeptide has its probability adjusted by abundance using the following formula:

$$P_{adjust,g} = P_{input} * e^{mean(abundance_g) - mean(abundance_{all})}$$

Wherein a glycopeptide, g, has a probability equal to the input probability multiplied by e raised to the power of the mean of the abundance of g less the mean of all glycopeptides in the dataset. Any glycopeptides that are adjusted to a probability greater than 1 are set to 0.99999999, and any that are adjusted to less than 0 are set to 0.00000001. If presence is set to 1, then the probability is not adjusted for any glycopeptide. This adjusted probability is then used in an binomial distribution to determine the number of samples that keep their abundances for that glycopeptide. The glycopeptide then has a random number of its remaining -if any- glycopeptides set to 0.

Outlier Detection

Modality Membership Proportion

Outlier Detection uses the Internal Similarity distributions and their modality. If an Internal Similarity Distribution is multimodal, peak membership can be determined by assessing the samplings that produced similarities between the troughs of each given peak. A leave one out z-score assessment is then used to determine over or under-representation in the peak with a cut off of greater than absolute z-score 3. Samples that are under-represented in the primary (highest similarity) peak or over-represented in secondary peaks are considered deleterious outliers and should be manually removed from the dataset by the user. Peaks that are over-represented in the primary peak or under-represented in secondary peaks are not deleterious outliers as they show higher consistency than the average sample.

Supplemental Results

Theoretical Examples

This section serves to show first how the theoretical data generator works. Then show different examples of comparisons. These figures are aimed at both proving validity and demonstrating use.

Supplemental Figure 2: Theoretical Data Generation

This graph shows the impacts of some of the modifications. The data in these plots are produced with default settings except for the means, the number of significant digits for the means, and the presence. The greater the number of significant digits, the less the variance becomes. Presence is also much more heavily affected in lower abundance values, which aligns with general expectations. The means were determined by 2 input vectors that are reverses of one another: 4E4,3.6E4,3.2E4,2.8E4,2.4E4,2E4,1.6E4,1.2E4,8E3,4E3. Generating the means: 0.0476…,0.142…,0.238…,0.333…,0.428…,0.523…,0.619…,0.714…,0.809…, and 0.904…

Figure S2: Theoretical Data: Variance vs Presence

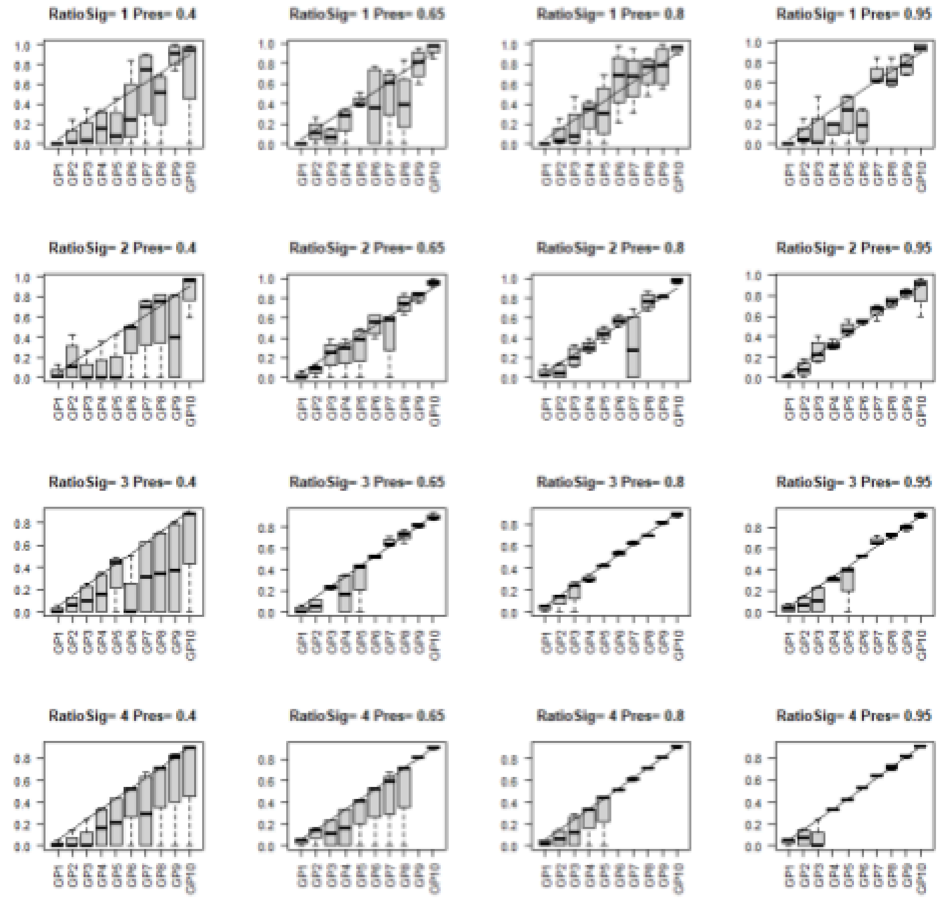

Theoretical Case: Same Underlying Distribution

Before proving that RAMZIS's similarity comparisons can find differences between groups, we must first prove that it finds groups with the same underlying glycosylation patterns to be similar.

For this, we generated a 12 glycopeptide dataset with 10 samples, low variance conditions (significant digits of mean=2, ratio multiplier=10^2 (Default=10^1)), and a presence of 1 with sampling means=c(0.0706,0.152,0.229,0.303,0.381,0.454,0.538,0.617,0.703,0.762,0.842,0.926) from initial alpha=c(400,800,1200,1600,2000,2400,2800,3200,3600,4000,4400,4800) and initial beta=c(4800,4400,4000,3600,3200,2800,2400,2000,1600,1200,800,400).

Then those 10 samples were split into two different datasets. This produced these abundances and similarity comparisons.

Supplemental Figure 3: Theoretical: Same Underlying

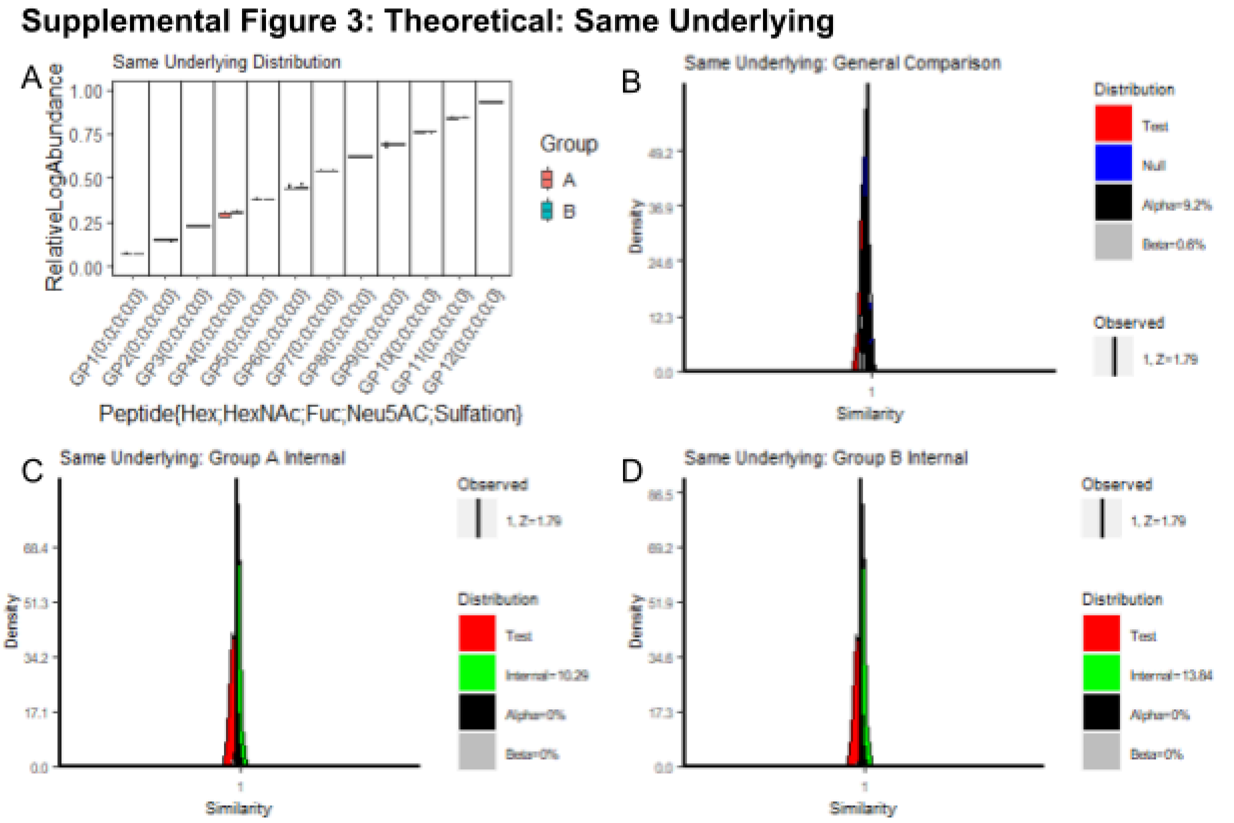

The abundances in S3a show very little variance between the groups. Figure S3b is the general comparison (between the Test and Null), which shows an FPR of 9.2% and FNR of 0.6%, exceeding the FPR threshold of 5%, so the two groups cannot be differentiated. The simulation of the comparison is within reliable ranges with an absolute z-score less than 2 at the Observed Similarity equal to 1 after rounding. We can say this confidently as seen in S3c and S3d where the Internal similarities are well over the confidence threshold and there is no substantial

overlap between them and the Test similarity distribution. With this example, we can confirm that RAMZIS won't find a spurious difference in the ideal circumstance. Note that the similarity plots displayed in S3 and S4 are shown with the x-axis confined to 0.9 to 1.1 for easier viewing.

RAMZIS Simulation Failure

To better resemble the abundances and variances seen in the AGP data in Figures 1a & 2d, we created another dataset with a decreased ratio exponent (10^1) compared to the prior dataset (10^2) to increase variability, and an initial alpha=c(1700,3550,3000,3660,6280,3720,3560,3920,3980,4480,6112,5000) and an initial beta of 2000 repeated 12 times; this produced sampling means=c(0.081,0.162,0.197,0.202,0.23,0.258,0.282,0.327,0.391,0.465,0.547,0.742) & expected variances=c(7.4e-06,2.7e-05,1.6e-05,3.2e-05,1.8e-4,3.8e-05,4e-05,2.2e-05,2.4e-05,1.2e-04,2.5e-05,3.8e-05) respectively. The generated abundances can be seen in S4a.

Supplemental Figure 4: Theoretical: Same Underlying Increased Variance

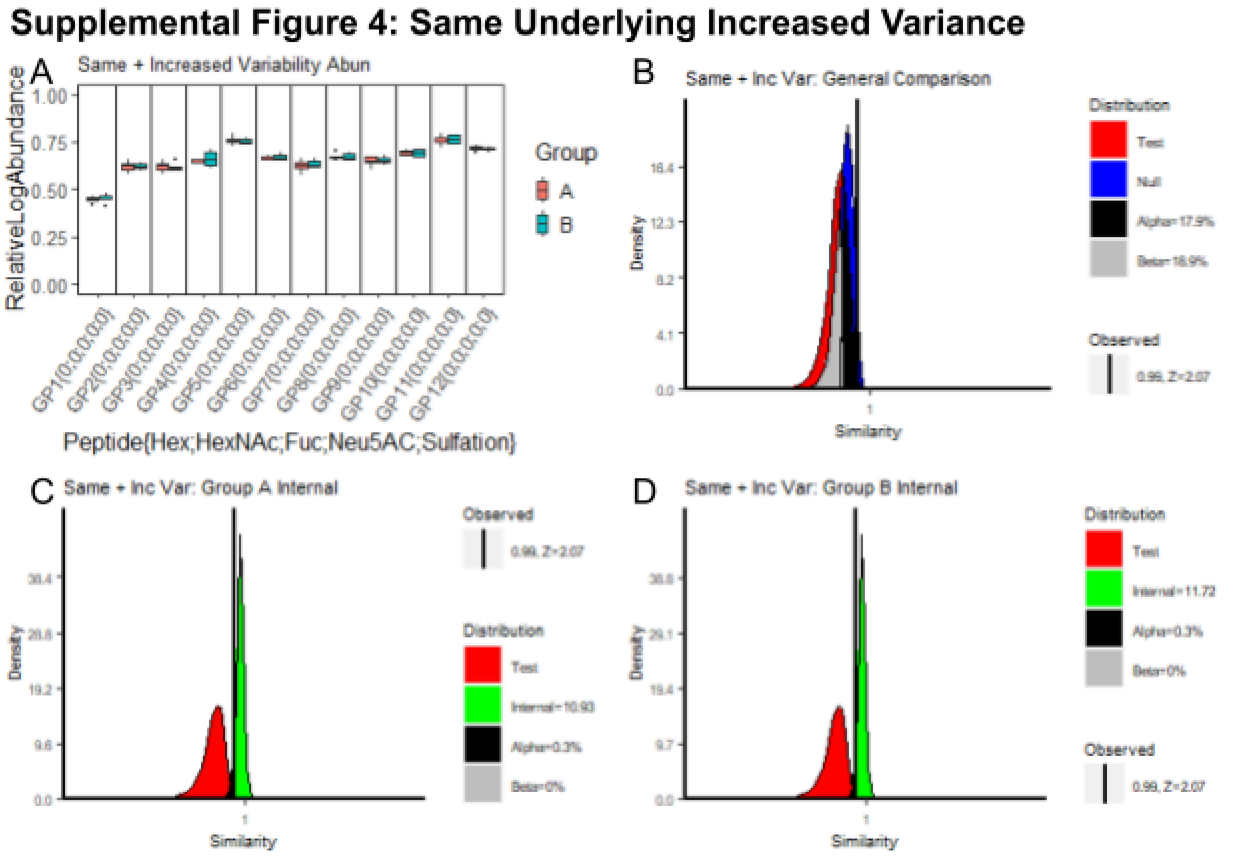

S4b shows that the increase in abundance doesn't produce a false positive differentiation in the Test vs Null general comparison with an FPR of 17.9% and an FNR of 18.9%, however it also shows a simulation failure. The Test distribution has the Observed similarity beyond acceptable bounds at 0.99 with a z-score of 2.07. This failure of simulation is increasingly likely in highly similar distributions as there is only so close to 1 that a similarity score can become. An

example of increased variance without simulation failure can be seen in S8 on the left hand column.

S4c and S4d show the Internal similarities of the two groups, and they both have high, tightly defined similarity distributions with high confidence scores. Less conservative approaches could put the z-score threshold at an absolute value of 3, but that would be an even less confident heuristic. While RAMZIS is not falsely differentiating datasets from the same underlying distribution here, it is ultimately because it cannot reliably simulate the comparison of two datasets that are so highly correlated, not because of the Test and Null overlap.
In fact, if we were to run Group A against itself in RAMZIS, as in Supplemental Figure 5, we see that RAMZIS fails once more in simulation as the observed similarity of the initial comparison is 1 as they are the exact same dataset.

Supplemental Figure 5: Theoretical: Group A vs Itself

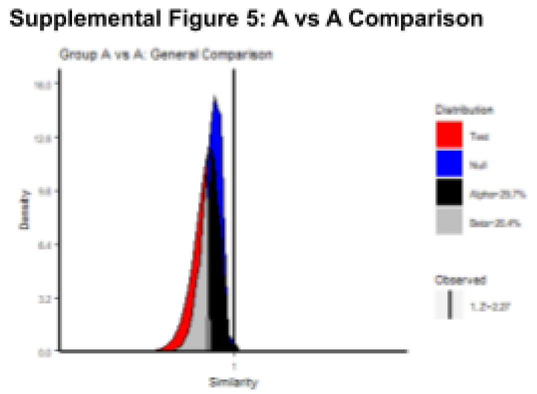

This simulation failure is a second fail safe among other quality checks. In highly consistent, highly similar datasets it can be easy to find small variations. If a dataset is well defined, then any variance from individual samples could falsely delineate the comparison. But if the samples are not outliers but are just not representative of specific glycopeptides, they will push the simulated Test comparisons to be lower similarities, so low that the Observed similarity can't be considered within its distribution. These glycopeptides can even be identified by the ranking information.

Ranking Elucidates Simulation Failure Causes

Supplementary Table 2 contains the Ranking Summary Table of the comparison found in Supplemental Figure 4, wherein we compared the more variable glycopeptides from the same underlying distribution.

Supplemental Table 2: Theoretical Rankings of Same + Inc

Glycopeptides	ZScore	PassOverall	FailCause
GP6	-0.32466	FALSE	LowQualityIn_Both
GP12	-0.23901	FALSE	LowQualityIn_Both
GP7	-0.22452	FALSE	LowQualityIn_Both
GP5	-0.21336	FALSE	LowQualityIn_Both
GP9	-0.20248	FALSE	LowQualityIn_Both
GP2	-0.18938	FALSE	LowQualityIn_Both

GP1	-0.18508	FALSE	LowQualityIn_Both
GP8	-0.12249	FALSE	LowQualityIn_Both
GP11	-0.09622	FALSE	LowQualityIn_Both
GP3	-0.08148	FALSE	LowQualityIn_Both
GP10	-0.07377	FALSE	LowQualityIn_Both
GP4	-0.05271	FALSE	LowQualityIn_Both

No glycopeptide is significantly separated here, but their rankings give us insight into the source of the Test similarities failure as a simulation. The two highest ranked glycopeptides are GP8 and GP2 can be seen to have visible outliers and wide variances respectively in Figure S4a. They both fail quality metrics due to low quality; GP8 fails only in B, as it has a wider variance than in A, while GP2 fails in both datasets due to their variance relative to the comparison.

The next 3 glycopeptides all pass Internal quality metrics, which may feel strange visually, as GP4 in B is seemingly the most variant glycopeptide quantitation in the entire dataset in S4a. What is unseen is that within that variance there are clusters. GP4 in B has the sample abundances 0.62, 0.61, 0.70, 0.71, and 0.66. Compared to GP2 in B with abundances of 0.63, 0.61, 0.58, 0.66, and 0.63, it is easy to identify that GP2 has a more uniform spread in its abundances, even if they are over a smaller area. The groupings of GP4, being farther from the overall average of GP4, will produce weighted Test similarity contributions that are substantially lower than those in its Internal Similarity as there will be other samplings of itself that aren't as far in average abundance to compare against. This separation provides the clarity that the Internal and Test behaviors are not the same, which allows the comparison of the Test against the Null. This presents a case wherein standard assessments of variability may not represent glycopeptide behaviors well as there is an assumption of sampling size that cannot be met in glycoproteomic experiments.

Theoretical Case: Completely Different

As a briefer demonstration of proof of concept, the comparison shown here is from two theoretical glycosylation patterns that are completely different. Supplemental Figure 6 shows the boxplot abundances in the top-right, the general comparison in the bottom left, and the internal similarities in the remaining corners. Group A has means=c(0.077,0.154,0.231,0.308,0.385,0.462,0.538,0.615,0.692,0.769,0.846,0.923) and group B has the same means but in reverse order; additionally group A has five samples while group B has ten. The glycopeptides are named GP1 to GP12 from left to right with the above corresponding means. RAMZIS can easily simulate this comparison as shown by the z-score of the observed in the Test of 0.11. The Internal similarities are both highly separated from the Test similarity with ICs of 114 and 404, and the Null is well separated from the Test with 0% overlap and thus 0% FNR and FPR.

The multimodality observed in the Null stems from the equal probability sampling of the two differently sized sample groups; while groups are equally likely to be observed, individual samples from the smaller group appear more frequently than individual samples from the larger group. This, in addition to the disparate means produced by these samplings, make multimodal effects in Null comparisons more likely to appear. While the effect size of this phenomenon is

not easily quantifiable, it is a qualitative data point that indicates the two sample groups do not share an underlying distribution.

Supplementary Table 3 holds the rankings for this comparison of completely different glycosylation patterns. The rankings here show that the rankings align with our intuitive understanding of the differences in this dataset. The highest ranked glycopeptides are those with the biggest differences between their means, GP1 and GP12. This trend holds true as the rankings descend from outermost to innermost, where there is the least difference between theoretical means. The quality assurance metrics show the conservative nature of these checks as seen in GP4 where the increase of variation was enough to identify it as a potential uncertainty, and in a real experiment, GP4 could be targeted in follow up experiments to discern its behavior if needed. Between GP4 and GP11, no glycosylation passes the z-score threshold for significant contributions to differences in similarity, further emphasizing the conservative nature of these recommendations. The z-score data is presented to the user for them to make their own assessment of the comparisons as they may wish to investigate edge cases like GPs 10 and 9, which both have between 2-3% chance of their mean coming from the Null contributions.

Supplemental Table 3: Theoretical Rankings of Completely Different

Glycopeptides	ZScore	PassOverall	FailCause
GP12	-2.612433122	TRUE	Passed
GP1	-2.581082019	TRUE	Passed
GP11	-2.529310851	TRUE	Passed
GP2	-2.471415015	TRUE	Passed
GP3	-2.370663323	TRUE	Passed
GP4	-2.240008857	FALSE	LowQualityIn_File1
GP10	-1.973772576	FALSE	LowQualityIn_File2
GP9	-1.958962841	TRUE	Passed
GP8	-1.875148407	TRUE	Passed
GP5	-1.663501443	FALSE	LowQualityIn_File1
GP6	-0.7712896788	TRUE	Passed
GP7	-0.5313232507	TRUE	Passed

Supplemental Figure 6: Theoretical: Completely Different

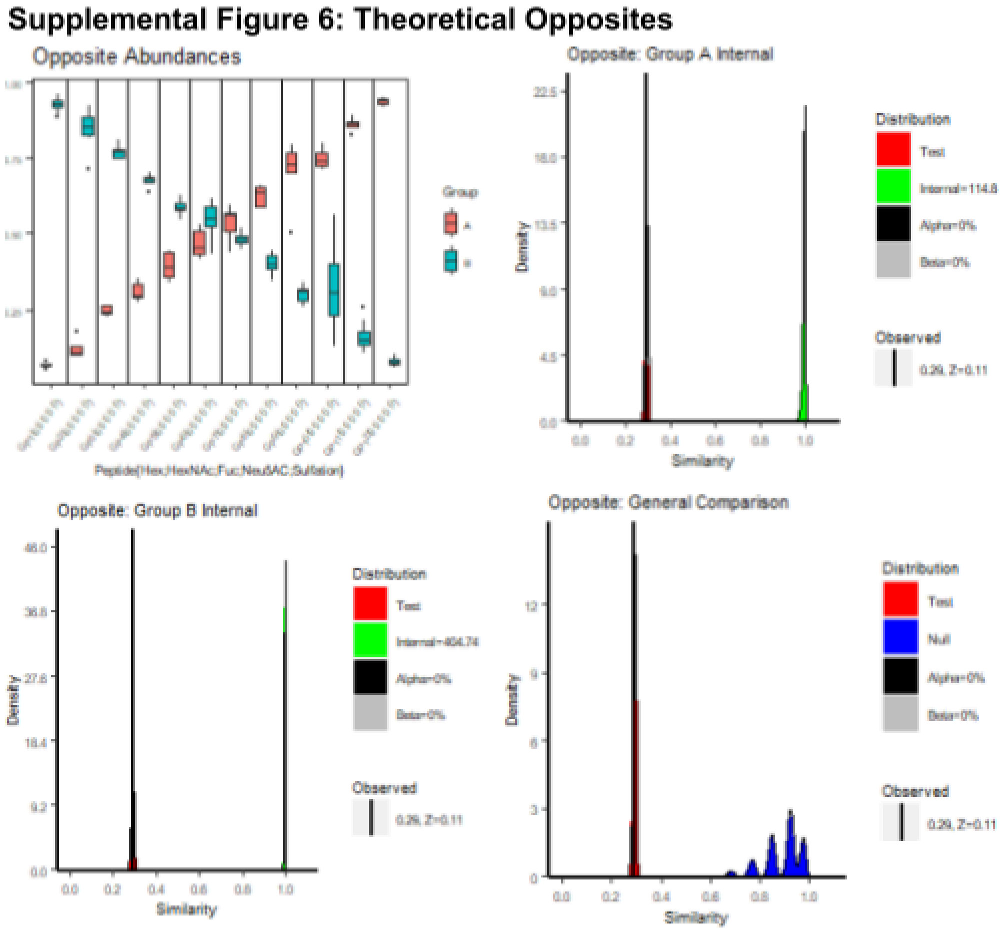

Theoretical Case: Major vs Minor Deviations

This section serves to show the effects of major and minor deviations on similarity comparisons. The major deviation is meant to show that RAMZIS does not wash out large individual shifts. The minor deviations section shows how small changes can lead to an overall shift in similarity.

Major Deviation

Using the same data as the Same Underlying example from Supplemental Figure 3, Supplemental Figure 7 shows the impact of a major deviation to one of the glycopeptides, GP9, wherein group B's GP9 has had its relative log abundance halved, which would indicate a fold change in relative log space and an even greater change in relative abundance space. All quality checks are passed; the Internal similarities are well separated and the observed is well simulated. There is no overlap between the Test and Null comparisons, and the Null is showing evidence of multimodality.

Supplemental Figure 7: Theoretical: Major Deviation

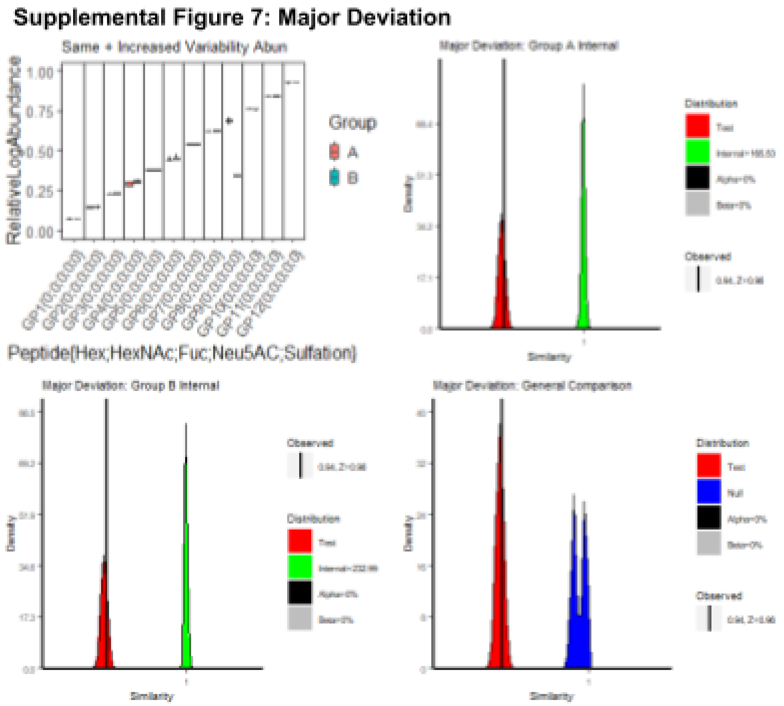

That presents the ideal case for a major deviation from an individual, a tightly defined glycopeptide undergoing a major change while remaining tightly defined, but that isn't representative of expected performance. The next example, Supplemental Figure 8, shows a major shift when variance is not as tightly defined; this data was generated by multiplying the tightly defined samples by different scalars from a normal distribution with mean of 1 and standard deviation of 0.05. The variability found here much better reflects that seen in real world cases, and we show that this increase in variability alone does not induce a false positive differentiation. The left hand side shows the increased variance without the GP9 deviation, while the right has the comparison after reducing GP9 in group B by 20%; these abundances can be seen in S8a.

Supplemental Figure 8: Theoretical: Major Deviation in Expected Variance

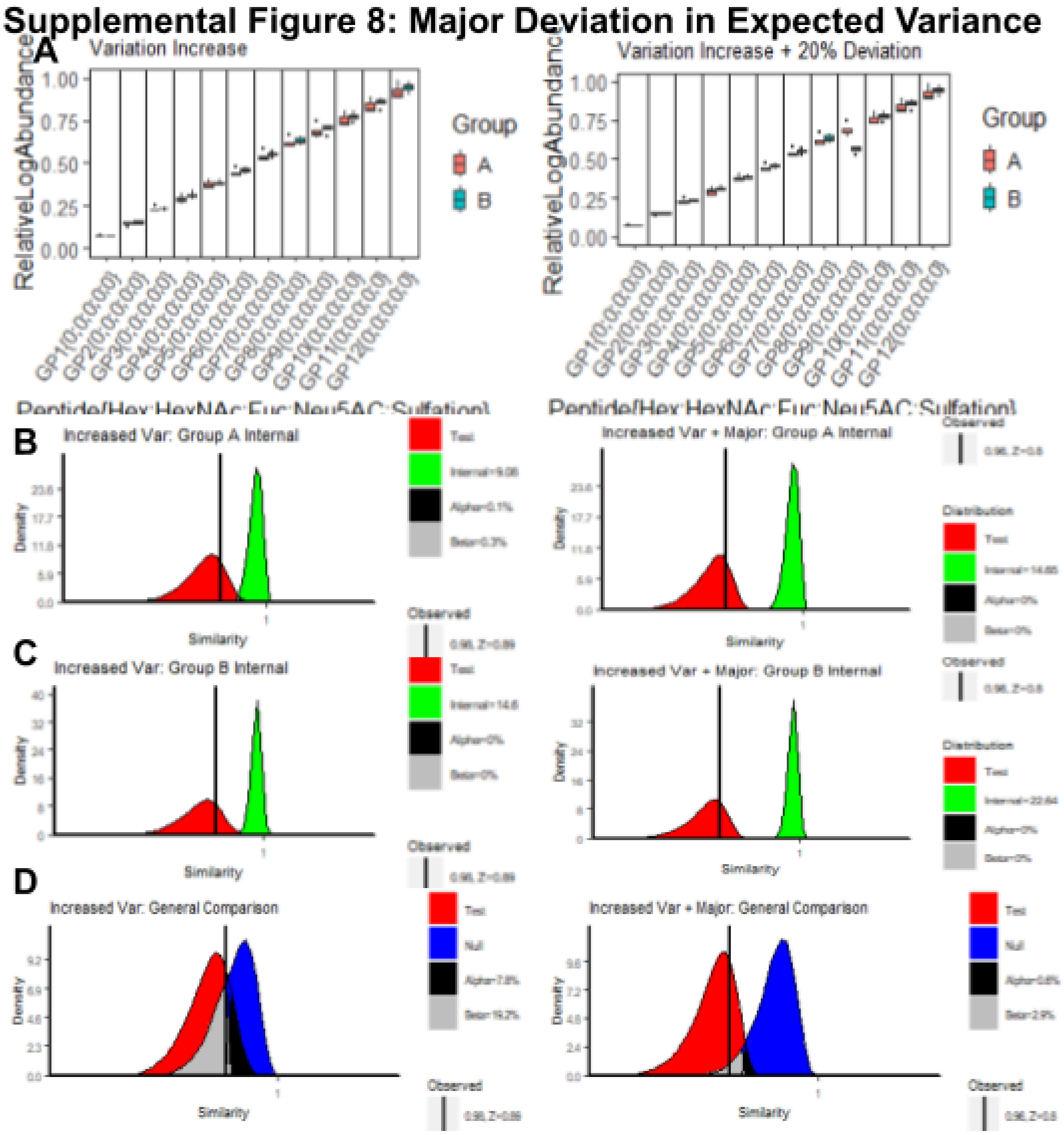

S8b and S8c show the Internal similarities which are well separated from the test in both scenarios with the same underlying having ICs of 9.08 and 14.6 while the deviation added scenario on the right has ICs of 14.65 and 22.64. Both Test similarities are well simulated with z-scores of 0.89 and 0.8 respectively, but while the left-hand scenario has too high an FPR (7.8%) to be differentiable, the right-hand (deviation added) scenario has an FPR of 0.6% and an FNR of 2.9%, allowing us to identify the difference in glycosylation pattern. The ranking for these are as expected with GP9 as the highest ranked in the deviation scenario with a z-score of -2.02 and a passing quality check; no other identifications passed quality or significance thresholds in either scenario. The 20% reduction for GP9 is actually near the edge of its detectable effect size; below this point, the glycosylation pattern change may be discernable, but GP9 no longer passes the -2 z-score threshold. 20% is not the minimum effect size for all identifications in this dataset, just the minimum for GP9. Minimum effect size is dependent on abundance magnitude and variation. As variation increases so too does minimum effect size. As

magnitude increases, minimum effect size decreases; our highest abundance identifications tend to be our most confident, and our least abundant tend to be our least confident identifications. This phenomenon makes it so that higher effect sizes are required to differentiate low confidence identifications.

Supplemental Figure 9: Minor Deviations: Overall changes without Individual significance

Minor deviations in this section refer to deviations that would each be under their minimal effect size normally. For the example in Supplemental Figure 9, the increased variance dataset from Supplemental Figure 8 was used, but in group B: GP9 was reduced by 15%, GP3 was reduced by 20%, and GP7 was increased by 10%. These shifts can be observed in S9a. None of these pass their minimum effect size for RAMZIS.

Supplemental Figure 9: Minor Deviations Lead to Global Change

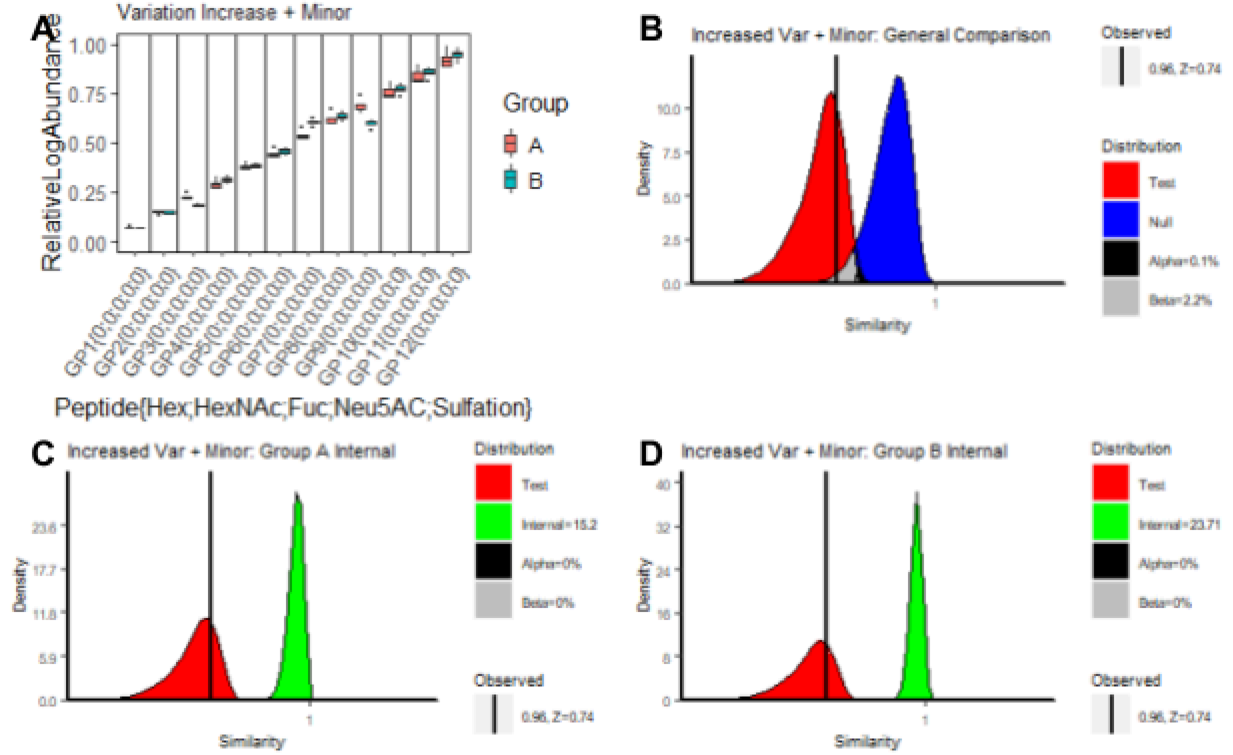

The Internal Similarities, shown in S9c and S9d for completeness, both have ICs (15.2 and 23.71 respectively) greater than 2, and the observed is well simulated by the Test with a z-score of 0.74. The general comparison of Test and Null in S9b shows minimal overlap with a 0.1% FPR and a 2.2% FNR, producing a similar level of confidence in the differentiation between Test and Null as was present in the Major deviation example in Supplemental Figure 8.

In the ranking information, shown in Supplementary Table 4, no identifications exceed significance thresholds as expected. This allows the user to conclude that the overall glycosylation pattern has changed, but individual glycopeptides have too subtle of changes to

be reliably discerned by RAMZIS. The highest ranked glycopeptides in this scenario make good candidates for follow up targeted experiments, or for more careful statistical analysis. Regardless of the individual results, the knowledge that there is a detectable disruption to the underlying glycosylation pattern for a glycosite innately informs the user to more closely investigate the discrepancies found.

Supplemental Table 4: Ranking Minor Deviations

Glycopeptides	ZScore	PassOverall	FailCause
GP9	-1.556410091	TRUE	Passed
GP7	-1.442600868	FALSE	LowQualityIn_File1
GP3	-0.8891118134	TRUE	Passed
GP12	-0.3878931912	FALSE	LowQualityIn_File1
GP6	-0.3522643834	FALSE	LowQualityIn_File1
GP11	-0.2977126149	FALSE	LowQualityIn_File1
GP10	-0.1721278478	FALSE	LowQualityIn_File1
GP4	-0.1426586041	FALSE	LowQualityIn_File1
GP8	-0.138142124	FALSE	LowQualityIn_Both
GP5	-0.09878190344	FALSE	LowQualityIn_Both
GP2	-0.03038656801	FALSE	LowQualityIn_Both
GP1	0.09365809483	FALSE	LowQualityIn_Both

Theoretical Case: Outlier Identification

RAMZIS identifies sample-wide outliers in sample groups; identification of glycopeptide specific outliers is not a default function of RAMZIS but could theoretically be done using a similar process. RAMZIS should identify samples with: increased rates of missingness, significant shifts in the mean glycosite abundance, mischaracterized samples, and true behavioral outliers. An example of an increased missingness outlier can be found below in the AGP Outlier Removal section at Supplemental Figure 11. RAMZIS does not identify what type of outliers it identifies as that requires manual interpretation, but it cannot identify outliers below a specific effect size. It is possible for a sample to be an outlier for one glycosite, but not others, and so the user should not blindly apply outlier removal without further examination. Effect size is directly correlated to RAMZIS' ability to identify outliers, and so subtle outliers may not be identified; individual glycosite outliers can only be identified in extreme cases.

Supplemental Figure 10: Theoretical: Outlier Removal

Here in Supplemental Figure 10 we present an example of a global shift in glycosite abundance for an individual sample. The data used is the increased variance data from Supplemental Figure 8 with group B's sample 1 reduced by 10%. Due to the high overall similarity, the default RAMZIS plotting functions were not used to illustrate this example.

S10a and S10d both contain Relative Log Abundance boxplots, S10a is with the 10% shifted sample while S10d is without it. While the effect size is limited, such that the difference is barely discernible in the plots, it is identifiable as shown in S10b and S10c. S10c is output text from RAMZISMain() that informs the user there is likely an outlier. That outlier is identified in S10b,

where the text output shows the outlier is the first sample, as it is indexed at [[1]], and that sample 1 is over-represented in the peak at 0.989 with a z-score of 18.7 and under-represented in the peak 0.996 with a z-score of -19.4. These peaks are plotted above this text output in a density plot of the Internal similarity of group B. The dashed lines are the under or over represented peaks in question while the solid lines are the peak minima. After removing the outlier sample in S10d, rerunning RAMZISMain(), we can then make the same kind of plot for S10e with the new comparison. We see that the plot is still multimodal, but the output indicates this comes from sample 4, and that the sample is under-represented in the second highest peak. While it is up to the user's discretion, this outlier was not removed as it was not under-represented in the highest, most-similar peak. This can be thought of as a positive outlier, a sample that is highly consistent. It is worth noting that the emergence of negative outliers, the kind that should be removed, after removal of a different outlier, may indicate the existence of a subgroup rather than a true outlier.

Supplemental Figure 10: Outlier Removal

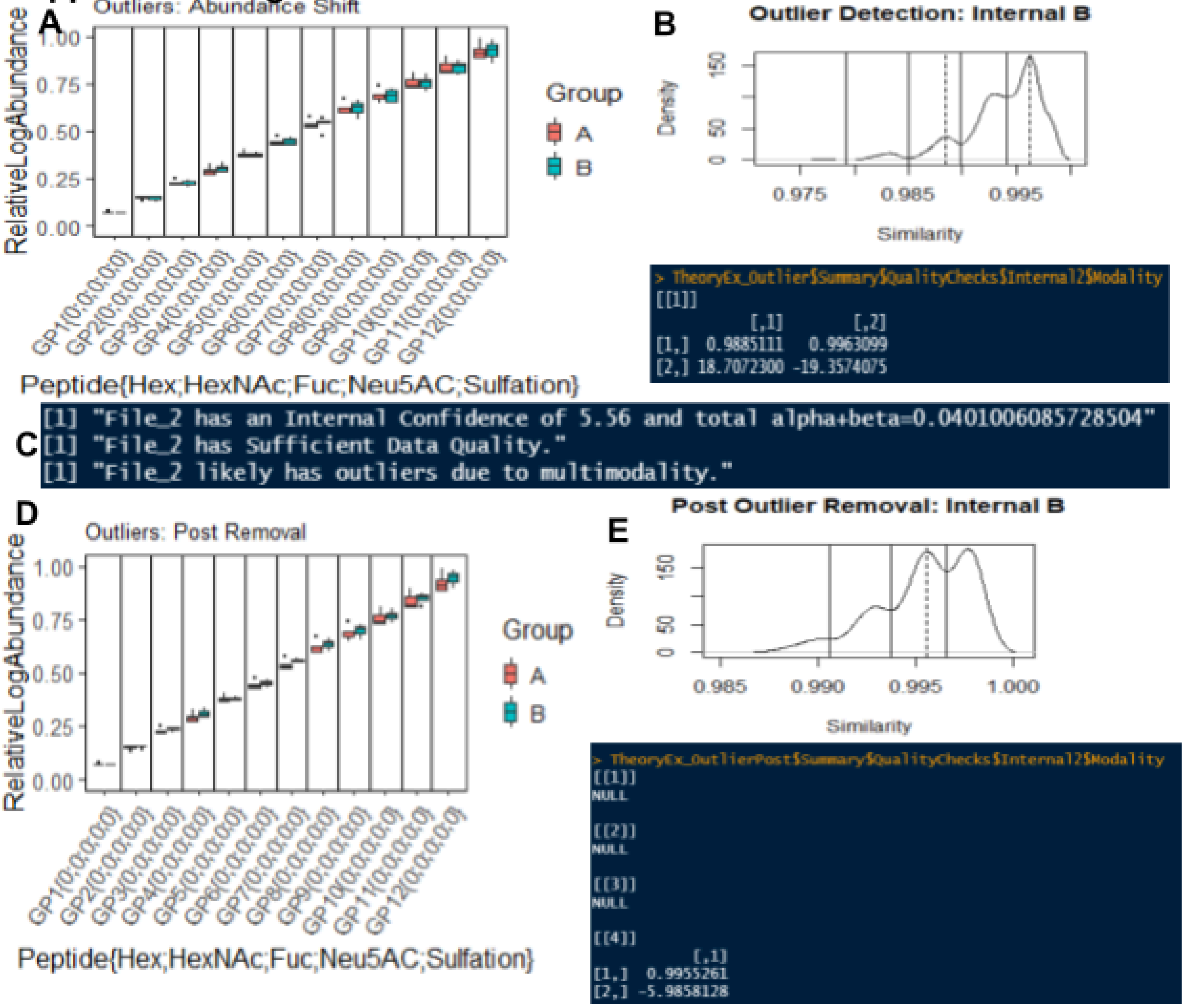

Manual examination of outliers is still critical with this tool. Even in this example, a manual examination of the outlier would reveal its uniform decrease in abundance. Ideally, this should

prompt the user to identify the source of this deviation. If it’s from a decrease in the protein level associated with that individual sample or if there is an overall decrease in the glycoproteomic signal in that sample, then the answer may not be outlier removal, but signal correction.

AGP Case-Study: Pure AGP

This section contains further elaboration on the analysis performed in the main text of the article. Outlier Removal refers to the identification and removal of an outlier sample in the Mix 1 AGP data. Full Ranking Summary contains the ranking data of the main-text analysis of Mix 1: Informed vs Naive.

Outlier Removal

In Mix 1, Informed vs Naive, there were originally four samples, but one of them was identified as an outlier as shown in Supplemental Figure 11. S11A shows the boxplot of relative log abundances, which shows a much higher degree of missingness compared to the outlier removed dataset in Figure 1A. Figure 1B shows the general comparison of Test and Null similarities, which show an FPR over the 5% threshold at 6%, and an FNR of 17.4%. Visually, the Test and Null similarities have substantially longer left-tails, which would account for much of the increased overlap. RAMZIS still successfully simulated this comparison with an observed similarity at a z-score of 1.25 in the Test similarity.

Supplemental Figure 11: AGP: Outlier Removal

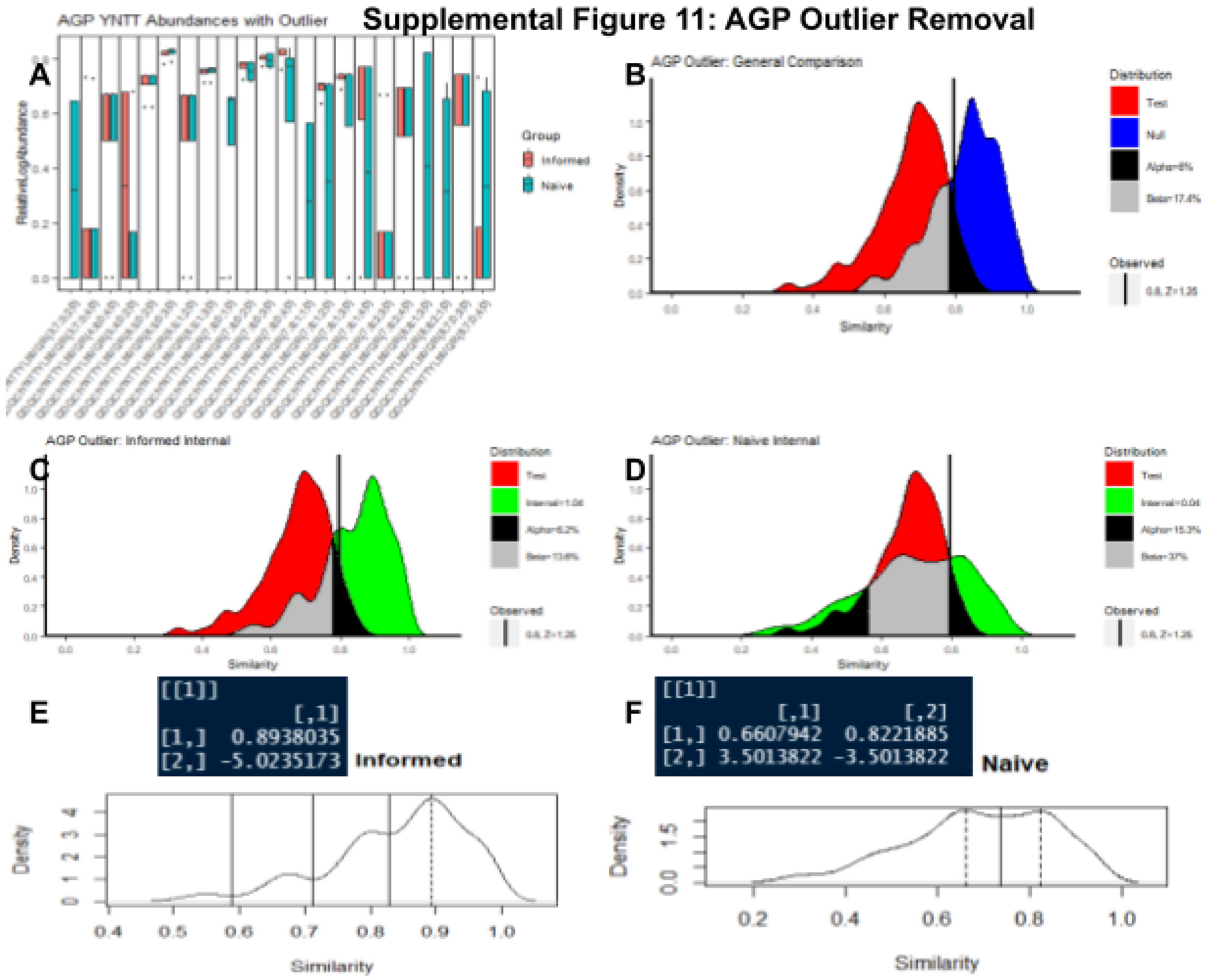

Figure S11C and S11D both show standard RAMZIS Internal similarity outputs, and both Naive and Informed have multimodality ongoing, though the Naive is not substantially different from its outlier removed comparison in Figure 1B. To help illustrate the outliers location, Figures S11E and S11F show the reduced plots found in S10, where the internal similarities are plotted on their own with the pertinent peaks marked with dashed lines and local minima marked with solid lines to show the boundaries of peak ownership. The RAMZIS text output has been overlaid with these plots to show the over and under representation in correspondence with the dashed peaks. In S11E, the Informed search has sample 1 (designated [[1]]) underrepresented (the second row z-score: -5.02) in the peak with a maxima at similarity at 0.894. This peak is the highest, most similar in the Informed Internal similarity, meaning that there are fewer observations of sample 1 in that peak than expected. S11F shows that the Naive search also has an under-representation (z-score=-3.501) in its most similar peak (0.822) and is over-represented (z=3.501) in its secondary peak (0.661).

This information shows that sample 1, in both searches, is an outlier in behavior, and is so in a deleterious way as it is under-represented in the most similar peaks of the Internal similarities.

Further analysis of Sample 1 showed that it had a higher rate of missingness. When it did observe glycopeptides, the abundances were not outside of leave-one-out variances, meaning that its outlier behavior is entirely from its missingness. The TIC from this run was substantially lower than the other run's TICs, indicating that there was likely a sample preparation or LC related error of some kind resulting in lower signal strength. In this case, the outlier sample had a TIC of 3.7E8 compared to TICs of 6.4E9, 6.3E9, and 4.9E9, making it a whole order of magnitude smaller. Lower signal strength is not the direct cause of the outlier removal, but a symptom; lower TICs are correlated with higher rates of missingness due to lower precursor acquisition rates and fewer product ions in individual spectra.

Full Ranking Summary

This section contains the full glycopeptide ranking table from the analysis performed in section 3.1.1: Naive vs Informed search spaces in pure AGP after outlier removal. This can be found in Supplemental Table 5.

Supplemental Table 5: Pure AGP Glycopeptide Rankings

Glycopeptides	ZScore	PassOverall	FailCause
QDQCIYNTTYLNVQR{Hex:7; HexNAc:6; Neu5Ac:1}	-1.946552562	FALSE	UnseenIn_File2
QDQCIYNTTYLNVQR{Hex:6; HexNAc:5; Neu5Ac:3}	-0.9344698552	TRUE	Passed
QDQCIYNTTYLNVQR{Fuc:1; Hex:7; HexNAc:6; Neu5Ac:1}	-0.8863841745	FALSE	LowQualityIn_Both
QDQCIYNTTYLNVQR{@sulfate:1; Fuc:5; Hex:3; HexNAc:7; Neu5Ac:2}	-0.8849453208	FALSE	LowQualityIn_Both
QDQCIYNTTYLNVQR{Fuc:1; Hex:8; HexNAc:6; Neu5Ac:3}	-0.880557342	FALSE	LowQualityIn_Both
QDQCIYNTTYLNVQR{Fuc:1; Hex:6; HexNAc:5; Neu5Ac:3}	-0.6147228118	TRUE	Passed
QDQCIYNTTYLNVQR{Hex:7; HexNAc:6; Neu5Ac:4}	-0.4792286414	TRUE	Passed
QDQCIYNTTYLNVQR{Hex:7; HexNAc:6; Neu5Ac:2}	-0.1444044722	TRUE	Passed
QDQCIYNTTYLNVQR{Hex:5; HexNAc:4; Neu5Ac:2}	-0.1323206534	FALSE	LowQualityIn_Both
QDQCIYNTTYLNVQR{Hex:8; HexNAc:7; Neu5Ac:4}	-0.1174572186	FALSE	LowQualityIn_Both
QDQCIYNTTYLNVQR{Hex:7; HexNAc:6; Neu5Ac:3}	-0.1106629259	TRUE	Passed
QDQCIYNTTYLNVQR{Fuc:1; Hex:7; HexNAc:6; Neu5Ac:2}	-0.09310602893	FALSE	LowQualityIn_File1
QDQCIYNTTYLNVQR{Fuc:1; Hex:7; HexNAc:6; Neu5Ac:4}	-0.09155135654	FALSE	LowQualityIn_File1
QDQCIYNTTYLNVQR{Fuc:1; Hex:7; HexNAc:6; Neu5Ac:3}	-0.05578959387	TRUE	Passed
QDQCIYNTTYLNVQR{Hex:6; HexNAc:5; Neu5Ac:2}	-0.05196458121	TRUE	Passed
QDQCIYNTTYLNVQR{Hex:8; HexNAc:7; Neu5Ac:3}	-0.04765218453	TRUE	Passed
QDQCIYNTTYLNVQR{@sulfate:1; Hex:4; HexNAc:6; Neu5Ac:4}	-0.04729701855	TRUE	Passed
QDQCIYNTTYLNVQR{Fuc:2; Hex:7; HexNAc:6; Neu5Ac:4}	-0.04723632322	TRUE	Passed
QDQCIYNTTYLNVQR{Fuc:1; Hex:6; HexNAc:5; Neu5Ac:2}	-0.04632468883	TRUE	Passed
QDQCIYNTTYLNVQR{Fuc:2; Hex:7; HexNAc:6; Neu5Ac:3}	-0.007826979993	FALSE	LowQualityIn_Both
QDQCIYNTTYLNVQR{@sulfate:1; Fuc:5; Hex:3; HexNAc:7; Neu5Ac:4}	-0.001148441625	FALSE	LowQualityIn_Both
QDQCIYNTTYLNVQR{@sulfate:1; Fuc:2; Hex:4; HexNAc:8; Neu5Ac:1}	NA	FALSE	BelowMinimumObs
QDQCIYNTTYLNVQR{@sulfate:1; Fuc:2; Hex:4; HexNAc:8; Neu5Ac:3}	NA	FALSE	BelowMinimumObs
QDQCIYNTTYLNVQR{@sulfate:1; Hex:4; HexNAc:6; Neu5Ac:3}	NA	FALSE	BelowMinimumObs
QDQCIYNTTYLNVQR{Fuc:1; Hex:6; HexNAc:4; Neu5Ac:1}	NA	FALSE	BelowMinimumObs
QDQCIYNTTYLNVQR{Fuc:1; Hex:8; HexNAc:6; Neu5Ac:1}	NA	FALSE	BelowMinimumObs
QDQCIYNTTYLNVQR{Fuc:1; Hex:8; HexNAc:6; Neu5Ac:2}	NA	FALSE	BelowMinimumObs
QDQCIYNTTYLNVQR{Fuc:1; Hex:8; HexNAc:7; Neu5Ac:4}	NA	FALSE	BelowMinimumObs
QDQCIYNTTYLNVQR{Fuc:2; Hex:8; HexNAc:6; Neu5Ac:1}	NA	FALSE	BelowMinimumObs
QDQCIYNTTYLNVQR{Fuc:2; Hex:8; HexNAc:6; Neu5Ac:3}	NA	FALSE	BelowMinimumObs
QDQCIYNTTYLNVQR{Hex:8; HexNAc:7; Neu5Ac:2}	NA	FALSE	BelowMinimumObs

AGP Case-Study: Mix 1 vs Mix 5

This section provides additional context to the analysis pertaining to section 3.1.2 and Figure 2, the comparisons of Mix 1 (pure AGP) and Mix 5 in both the Naive and Informed comparisons with respect to glycosite YnTT.

Naive Scaled

The rankings in Supplemental Table 6 pertain to the scaled analysis done in 3.1.2. Scaling was done to account for a global shift in abundance observed in Mix 5. Naive Mix 5 was scaled by 1.067.

Full Ranking Summary

Supplemental Table 6: Naive: Mix 1 vs Mix 5 Scaled

Glycopeptides	ZScore	PassOverall	FailCause
QDQCIYNTTYLNVQR{Hex:7; HexNAc:6; Neu5Ac:1}	-2.017354535	FALSE	UnseenIn_File2
QDQCIYNTTYLNVQR{Fuc:1; Hex:6; HexNAc:5; Neu5Ac:2}	-2.01515825	FALSE	UnseenIn_File2
QDQCIYNTTYLNVQR{@sulfate:1; Hex:4; HexNAc:6; Neu5Ac:4}	-2.014661812	FALSE	UnseenIn_File2
QDQCIYNTTYLNVQR{Fuc:2; Hex:7; HexNAc:6; Neu5Ac:4}	-2.00870877	FALSE	UnseenIn_File2
QDQCIYNTTYLNVQR{Fuc:1; Hex:7; HexNAc:6; Neu5Ac:3}	-2.000440825	FALSE	UnseenIn_File2
QDQCIYNTTYLNVQR{Hex:8; HexNAc:7; Neu5Ac:3}	-1.998416057	FALSE	UnseenIn_File2
QDQCIYNTTYLNVQR{Fuc:1; Hex:6; HexNAc:5; Neu5Ac:3}	-1.994785183	FALSE	UnseenIn_File2
QDQCIYNTTYLNVQR{@sulfate:1; Hex:4; HexNAc:8; Neu5Ac:3}	-1.993957069	FALSE	UnseenIn_File1
QDQCIYNTTYLNVQR{Hex:6; HexNAc:5; Neu5Ac:3}	-1.000902286	FALSE	LowQualityIn_File1
QDQCIYNTTYLNVQR{Hex:6; HexNAc:5; Neu5Ac:2}	-0.9274038643	FALSE	LowQualityIn_File1
QDQCIYNTTYLNVQR{Fuc:1; Hex:7; HexNAc:6; Neu5Ac:1}	-0.8863143119	FALSE	LowQualityIn_Both
QDQCIYNTTYLNVQR{@sulfate:1; Fuc:5; Hex:3; HexNAc:7; Neu5Ac:2}	-0.8849555986	FALSE	LowQualityIn_Both
QDQCIYNTTYLNVQR{@sulfate:1; Fuc:5; Hex:3; HexNAc:7; Neu5Ac:3}	-0.8794608999	FALSE	LowQualityIn_Both
QDQCIYNTTYLNVQR{@sulfate:1; Fuc:1; Hex:4; HexNAc:8; Neu5Ac:3}	-0.8792374146	FALSE	LowQualityIn_Both
QDQCIYNTTYLNVQR{@sulfate:1; Fuc:1; Hex:5; HexNAc:8; Neu5Ac:2}	-0.8790271722	FALSE	LowQualityIn_Both
QDQCIYNTTYLNVQR{Fuc:1; Hex:7; HexNAc:6; Neu5Ac:2}	-0.8566172218	FALSE	LowQualityIn_Both
QDQCIYNTTYLNVQR{Hex:7; HexNAc:6; Neu5Ac:3}	-0.5715212188	FALSE	LowQualityIn_File2
QDQCIYNTTYLNVQR{Fuc:1; Hex:8; HexNAc:6; Neu5Ac:3}	-0.1200502352	FALSE	LowQualityIn_Both
QDQCIYNTTYLNVQR{Hex:7; HexNAc:6; Neu5Ac:2}	-0.1054248179	FALSE	LowQualityIn_File1
QDQCIYNTTYLNVQR{Hex:7; HexNAc:6; Neu5Ac:4}	-0.07346798053	FALSE	LowQualityIn_File2
QDQCIYNTTYLNVQR{Fuc:2; Hex:8; HexNAc:6; Neu5Ac:3}	-0.0112437359	FALSE	LowQualityIn_Both
QDQCIYNTTYLNVQR{Fuc:1; Hex:6; HexNAc:4; Neu5Ac:1}	0.0006557152411	FALSE	LowQualityIn_Both
QDQCIYNTTYLNVQR{Fuc:1; Hex:7; HexNAc:6; Neu5Ac:4}	0.007203044013	FALSE	LowQualityIn_Both
QDQCIYNTTYLNVQR{Hex:8; HexNAc:7; Neu5Ac:4}	0.009189220675	FALSE	LowQualityIn_Both
QDQCIYNTTYLNVQR{@sulfate:1; Fuc:1; Hex:3; HexNAc:7; Neu5Ac:3}	NA	FALSE	BelowMinimumObs
QDQCIYNTTYLNVQR{@sulfate:1; Fuc:2; Hex:4; HexNAc:7; Neu5Ac:2}	NA	FALSE	BelowMinimumObs
QDQCIYNTTYLNVQR{@sulfate:1; Fuc:2; Hex:4; HexNAc:8; Neu5Ac:1}	NA	FALSE	BelowMinimumObs
QDQCIYNTTYLNVQR{@sulfate:1; Fuc:2; Hex:5; HexNAc:8; Neu5Ac:2}	NA	FALSE	BelowMinimumObs
QDQCIYNTTYLNVQR{@sulfate:1; Fuc:5; Hex:3; HexNAc:7; Neu5Ac:4}	NA	FALSE	BelowMinimumObs
QDQCIYNTTYLNVQR{@sulfate:1; Hex:10; HexNAc:5; Neu5Ac:1}	NA	FALSE	BelowMinimumObs
QDQCIYNTTYLNVQR{@sulfate:1; Hex:3; HexNAc:7; Neu5Ac:3}	NA	FALSE	BelowMinimumObs
QDQCIYNTTYLNVQR{@sulfate:1; Hex:4; HexNAc:6; Neu5Ac:3}	NA	FALSE	BelowMinimumObs
QDQCIYNTTYLNVQR{Fuc:1; Hex:8; HexNAc:6; Neu5Ac:1}	NA	FALSE	BelowMinimumObs
QDQCIYNTTYLNVQR{Fuc:1; Hex:8; HexNAc:6; Neu5Ac:2}	NA	FALSE	BelowMinimumObs
QDQCIYNTTYLNVQR{Fuc:1; Hex:8; HexNAc:7; Neu5Ac:4}	NA	FALSE	BelowMinimumObs
QDQCIYNTTYLNVQR{Fuc:2; Hex:7; HexNAc:5; Neu5Ac:2}	NA	FALSE	BelowMinimumObs
QDQCIYNTTYLNVQR{Fuc:2; Hex:7; HexNAc:6; Neu5Ac:3}	NA	FALSE	BelowMinimumObs

QDQCIYNTTYLNVQR{Fuc:2; Hex:8; HexNAc:6; Neu5Ac:1}	NA	FALSE	BelowMinimumObs
QDQCIYNTTYLNVQR{Fuc:2; Hex:8; HexNAc:6; Neu5Ac:2}	NA	FALSE	BelowMinimumObs
QDQCIYNTTYLNVQR{Hex:5; HexNAc:4; Neu5Ac:2}	NA	FALSE	BelowMinimumObs
QDQCIYNTTYLNVQR{Hex:8; HexNAc:7; Neu5Ac:2}	NA	FALSE	BelowMinimumObs

Naive Unscaled

Similarity Analysis

For completeness, the unscaled analysis is provided here in brief in Supplemental Figure 12. S12A contains the abundance boxplot, where there is a pronounced scalar shift. S12B and S12C show the Internal similarities of Mix 1 and 5 respectively which have ICs of 4.1 and 1.63 respectively. The observed similarity is well-simulated with a z-score of 0.87, and there is a clear separation between the Test and Null similarities as seen in S12D, the general comparison of Test and Null. S12E shows the linear model used to determine the scaling value used in the main text analysis. None of this analysis substantially differs from the Naive analysis of the main text.

Supplemental Figure 12: AGP: Naive Unscaled: Mix 1 vs Mix 5

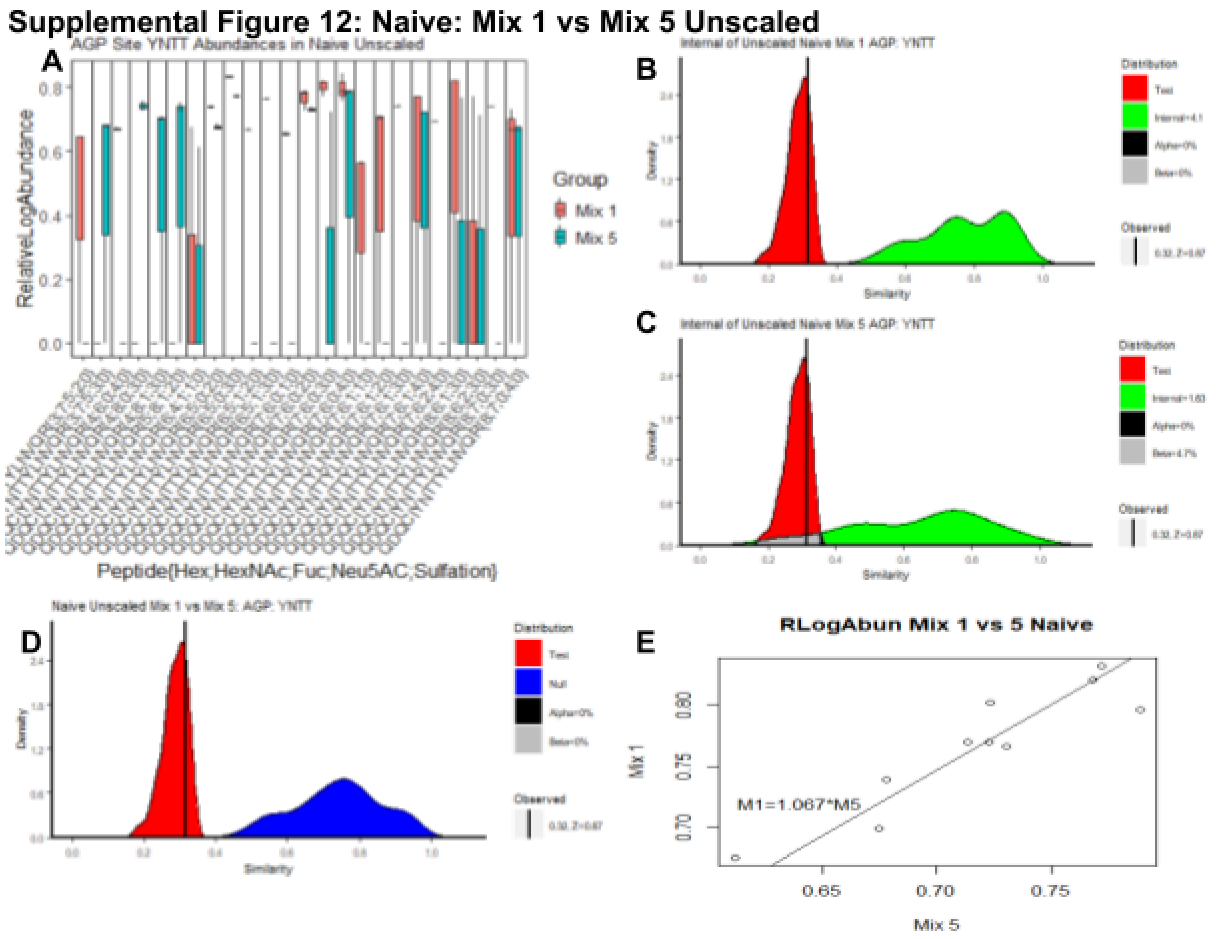

Full Ranking Summary

For completeness, the rankings of the unscaled Naive analysis are provided here. The order of glycopeptides is shifted, and several z-scores have dropped below significance, but the primary cause of differentiation in the highest ranked contributors to dissimilarity is the same as the scaled: disjoint observations between the two sample groups.

Supplemental Table 7: Naive: Mix 1 vs Mix 5 Unscaled

Glycopeptides	ZScore	PassOverall	FailCause
QDQCIYNTTYLNVQR{Hex:7; HexNAc:6; Neu5Ac:1}	-1.978072065	FALSE	UnseenIn_File2
QDQCIYNTTYLNVQR{Fuc:1; Hex:6; HexNAc:5; Neu5Ac:2}	-1.974507167	FALSE	UnseenIn_File2
QDQCIYNTTYLNVQR{@sulfate:1; Hex:4; HexNAc:6; Neu5Ac:4}	-1.973847799	FALSE	UnseenIn_File2
QDQCIYNTTYLNVQR{Fuc:2; Hex:7; HexNAc:6; Neu5Ac:4}	-1.966647442	FALSE	UnseenIn_File2
QDQCIYNTTYLNVQR{@sulfate:1; Hex:4; HexNAc:8; Neu5Ac:3}	-1.960116619	FALSE	UnseenIn_File1
QDQCIYNTTYLNVQR{Fuc:1; Hex:7; HexNAc:6; Neu5Ac:3}	-1.958942465	FALSE	UnseenIn_File2
QDQCIYNTTYLNVQR{Hex:8; HexNAc:7; Neu5Ac:3}	-1.957859691	FALSE	UnseenIn_File2
QDQCIYNTTYLNVQR{Fuc:1; Hex:6; HexNAc:5; Neu5Ac:3}	-1.95526794	FALSE	UnseenIn_File2
QDQCIYNTTYLNVQR{Hex:6; HexNAc:5; Neu5Ac:3}	-1.831499753	FALSE	LowQualityIn_File1
QDQCIYNTTYLNVQR{Hex:6; HexNAc:5; Neu5Ac:2}	-1.596834036	FALSE	LowQualityIn_File1
QDQCIYNTTYLNVQR{@sulfate:1; Fuc:1; Hex:5; HexNAc:8; Neu5Ac:2}	-0.8931595811	FALSE	LowQualityIn_Both
QDQCIYNTTYLNVQR{@sulfate:1; Fuc:1; Hex:4; HexNAc:8; Neu5Ac:3}	-0.8926321709	FALSE	LowQualityIn_Both
QDQCIYNTTYLNVQR{@sulfate:1; Fuc:5; Hex:3; HexNAc:7; Neu5Ac:3}	-0.8923796016	FALSE	LowQualityIn_Both
QDQCIYNTTYLNVQR{Fuc:1; Hex:7; HexNAc:6; Neu5Ac:2}	-0.8826370706	FALSE	LowQualityIn_Both
QDQCIYNTTYLNVQR{Fuc:1; Hex:7; HexNAc:6; Neu5Ac:1}	-0.8348766778	FALSE	LowQualityIn_Both
QDQCIYNTTYLNVQR{@sulfate:1; Fuc:5; Hex:3; HexNAc:7; Neu5Ac:2}	-0.8333030862	FALSE	LowQualityIn_Both
QDQCIYNTTYLNVQR{Hex:7; HexNAc:6; Neu5Ac:3}	-0.6642829836	FALSE	LowQualityIn_File2
QDQCIYNTTYLNVQR{Hex:7; HexNAc:6; Neu5Ac:2}	-0.545866928	FALSE	LowQualityIn_File1
QDQCIYNTTYLNVQR{Fuc:1; Hex:8; HexNAc:6; Neu5Ac:3}	-0.1719883895	FALSE	LowQualityIn_Both
QDQCIYNTTYLNVQR{Hex:7; HexNAc:6; Neu5Ac:4}	-0.1044163703	FALSE	LowQualityIn_File2
QDQCIYNTTYLNVQR{Fuc:1; Hex:6; HexNAc:4; Neu5Ac:1}	-0.01235929583	FALSE	LowQualityIn_Both
QDQCIYNTTYLNVQR{Fuc:2; Hex:8; HexNAc:6; Neu5Ac:3}	-0.01192585312	FALSE	LowQualityIn_Both
QDQCIYNTTYLNVQR{Fuc:1; Hex:7; HexNAc:6; Neu5Ac:4}	-0.008072765172	FALSE	LowQualityIn_Both
QDQCIYNTTYLNVQR{Hex:8; HexNAc:7; Neu5Ac:4}	0.001870856783	FALSE	LowQualityIn_Both
QDQCIYNTTYLNVQR{@sulfate:1; Fuc:1; Hex:3; HexNAc:7; Neu5Ac:3}	NA	FALSE	BelowMinimumObs
QDQCIYNTTYLNVQR{@sulfate:1; Fuc:2; Hex:4; HexNAc:7; Neu5Ac:2}	NA	FALSE	BelowMinimumObs
QDQCIYNTTYLNVQR{@sulfate:1; Fuc:2; Hex:4; HexNAc:8; Neu5Ac:1}	NA	FALSE	BelowMinimumObs
QDQCIYNTTYLNVQR{@sulfate:1; Fuc:2; Hex:5; HexNAc:8; Neu5Ac:2}	NA	FALSE	BelowMinimumObs
QDQCIYNTTYLNVQR{@sulfate:1; Fuc:5; Hex:3; HexNAc:7; Neu5Ac:4}	NA	FALSE	BelowMinimumObs
QDQCIYNTTYLNVQR{@sulfate:1; Hex:10; HexNAc:5; Neu5Ac:1}	NA	FALSE	BelowMinimumObs
QDQCIYNTTYLNVQR{@sulfate:1; Hex:3; HexNAc:7; Neu5Ac:3}	NA	FALSE	BelowMinimumObs
QDQCIYNTTYLNVQR{@sulfate:1; Hex:4; HexNAc:6; Neu5Ac:3}	NA	FALSE	BelowMinimumObs
QDQCIYNTTYLNVQR{Fuc:1; Hex:8; HexNAc:6; Neu5Ac:1}	NA	FALSE	BelowMinimumObs
QDQCIYNTTYLNVQR{Fuc:1; Hex:8; HexNAc:6; Neu5Ac:2}	NA	FALSE	BelowMinimumObs
QDQCIYNTTYLNVQR{Fuc:1; Hex:8; HexNAc:7; Neu5Ac:4}	NA	FALSE	BelowMinimumObs
QDQCIYNTTYLNVQR{Fuc:2; Hex:7; HexNAc:5; Neu5Ac:2}	NA	FALSE	BelowMinimumObs
QDQCIYNTTYLNVQR{Fuc:2; Hex:7; HexNAc:6; Neu5Ac:3}	NA	FALSE	BelowMinimumObs
QDQCIYNTTYLNVQR{Fuc:2; Hex:8; HexNAc:6; Neu5Ac:1}	NA	FALSE	BelowMinimumObs
QDQCIYNTTYLNVQR{Fuc:2; Hex:8; HexNAc:6; Neu5Ac:2}	NA	FALSE	BelowMinimumObs
QDQCIYNTTYLNVQR{Hex:5; HexNAc:4; Neu5Ac:2}	NA	FALSE	BelowMinimumObs
QDQCIYNTTYLNVQR{Hex:8; HexNAc:7; Neu5Ac:2}	NA	FALSE	BelowMinimumObs

Informed

The rankings in Supplemental Table 8 pertain to the scaled analysis done in 3.1.2. Scaling was done to account for a global shift in abundance observed in Mix 5. Informed Mix 5 was scaled by 1.084.

Full Ranking Summary

Supplemental Table 8: Informed: Mix 1 vs Mix 5 Scaled

Glycopeptides	ZScore	PassOverall	FailCause
QDQCIYNTTYLNVQR{Fuc:1; Hex:6; HexNAc:5; Neu5Ac:2}	-2.016914538	FALSE	UnseenIn_File2
QDQCIYNTTYLNVQR{@sulfate:1; Hex:4; HexNAc:6; Neu5Ac:4}	-2.016374361	FALSE	UnseenIn_File2
QDQCIYNTTYLNVQR{Fuc:1; Hex:7; HexNAc:6; Neu5Ac:2}	-2.011076058	FALSE	UnseenIn_File2
QDQCIYNTTYLNVQR{Fuc:2; Hex:7; HexNAc:6; Neu5Ac:4}	-2.007856868	FALSE	UnseenIn_File2
QDQCIYNTTYLNVQR{Fuc:1; Hex:7; HexNAc:6; Neu5Ac:3}	-2.000628783	FALSE	UnseenIn_File2
QDQCIYNTTYLNVQR{Hex:8; HexNAc:7; Neu5Ac:3}	-1.998418323	FALSE	UnseenIn_File2
QDQCIYNTTYLNVQR{@sulfate:1; Fuc:1; Hex:3; HexNAc:7; Neu5Ac:3}	-1.996835746	FALSE	UnseenIn_File1
QDQCIYNTTYLNVQR{@sulfate:1; Fuc:1; Hex:4; HexNAc:8; Neu5Ac:3}	-1.9952286	FALSE	UnseenIn_File1
QDQCIYNTTYLNVQR{@sulfate:1; Hex:4; HexNAc:8; Neu5Ac:3}	-1.987394794	FALSE	UnseenIn_File1
QDQCIYNTTYLNVQR{Hex:6; HexNAc:5; Neu5Ac:3}	-1.132543698	TRUE	Passed
QDQCIYNTTYLNVQR{Fuc:1; Hex:6; HexNAc:5; Neu5Ac:3}	-0.8417743956	FALSE	LowQualityIn_File2
QDQCIYNTTYLNVQR{Hex:7; HexNAc:6; Neu5Ac:2}	-0.6213296245	TRUE	Passed
QDQCIYNTTYLNVQR{Hex:7; HexNAc:6; Neu5Ac:3}	-0.5665884601	FALSE	LowQualityIn_File2
QDQCIYNTTYLNVQR{Fuc:1; Hex:7; HexNAc:6; Neu5Ac:4}	-0.4973306375	FALSE	LowQualityIn_File2
QDQCIYNTTYLNVQR{Hex:7; HexNAc:6; Neu5Ac:4}	-0.3426218304	TRUE	Passed
QDQCIYNTTYLNVQR{Hex:6; HexNAc:5; Neu5Ac:2}	-0.2106545612	TRUE	Passed
QDQCIYNTTYLNVQR{Hex:5; HexNAc:4; Neu5Ac:2}	-0.1396757738	FALSE	LowQualityIn_Both
QDQCIYNTTYLNVQR{@sulfate:1; Fuc:2; Hex:4; HexNAc:8; Neu5Ac:3}	NA	FALSE	BelowMinimumObs
QDQCIYNTTYLNVQR{@sulfate:1; Fuc:5; Hex:3; HexNAc:7; Neu5Ac:3}	NA	FALSE	BelowMinimumObs
QDQCIYNTTYLNVQR{@sulfate:1; Fuc:5; Hex:3; HexNAc:7; Neu5Ac:4}	NA	FALSE	BelowMinimumObs
QDQCIYNTTYLNVQR{@sulfate:1; Hex:3; HexNAc:7; Neu5Ac:3}	NA	FALSE	BelowMinimumObs
QDQCIYNTTYLNVQR{Fuc:2; Hex:7; HexNAc:6; Neu5Ac:3}	NA	FALSE	BelowMinimumObs
QDQCIYNTTYLNVQR{Hex:8; HexNAc:7; Neu5Ac:4}	NA	FALSE	BelowMinimumObs

Informed Unscaled

Similarity Analysis

For completeness, the unscaled Informed analysis is provided in Supplemental Figure 13. S13A contains the abundance boxplot, where there is a pronounced scalar shift, more apparent here than in S12A. S13B and S13C show the Internal similarities of Mix 1 and 5 respectively which have ICs of 12.63 and 5.81 respectively. The observed similarity is well-simulated with a z-score of 0.1, and there is a clear separation between the Test and Null similarities as seen in S13D, the general comparison of Test and Null for unscaled Informed. S13E shows the linear model used to determine the scaling value used in the main text analysis. None of this analysis substantially differs from the Informed analysis of the main text.

Supplemental Figure 13: AGP: Informed Unscaled: Mix 1 vs Mix 5

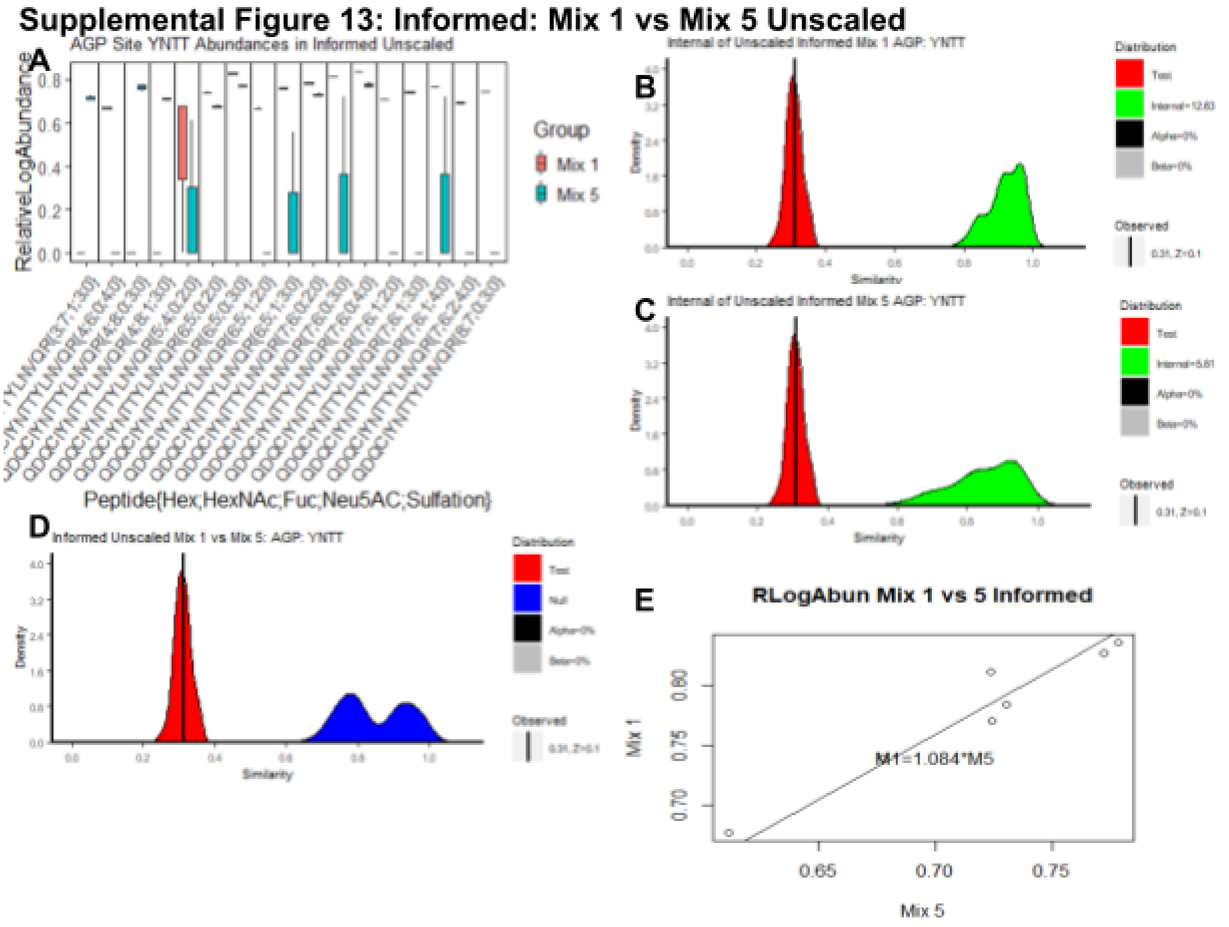

Full Ranking Summary

For completeness, the rankings of the unscaled Informed analysis are provided here. The order of glycopeptides is shifted, and several z-scores have dropped below significance, but the primary cause of differentiation in the highest ranked contributors to dissimilarity is once again the same as the scaled analysis: disjoint observations between the two sample groups.

Supplemental Table 9: Informed: Mix 1 vs Mix 5 Unscaled

Glycopeptides	ZScore	PassOverall	FailCause
QDQCIYNTTYLNVQR{Fuc:1; Hex:6; HexNAc:5; Neu5Ac:2}	-1.949874116	FALSE	UnseenIn_File2
QDQCIYNTTYLNVQR{@sulfate:1; Hex:4; HexNAc:6; Neu5Ac:4}	-1.94942564	FALSE	UnseenIn_File2
QDQCIYNTTYLNVQR{@sulfate:1; Fuc:1; Hex:3; HexNAc:7; Neu5Ac:3}	-1.945930146	FALSE	UnseenIn_File1
QDQCIYNTTYLNVQR{Fuc:2; Hex:7; HexNAc:6; Neu5Ac:4}	-1.944599675	FALSE	UnseenIn_File2
QDQCIYNTTYLNVQR{@sulfate:1; Fuc:1; Hex:4; HexNAc:8; Neu5Ac:3}	-1.943812194	FALSE	UnseenIn_File1
QDQCIYNTTYLNVQR{Fuc:1; Hex:7; HexNAc:6; Neu5Ac:2}	-1.943716071	FALSE	UnseenIn_File2
QDQCIYNTTYLNVQR{@sulfate:1; Hex:4; HexNAc:8; Neu5Ac:3}	-1.939871946	FALSE	UnseenIn_File1
QDQCIYNTTYLNVQR{Fuc:1; Hex:7; HexNAc:6; Neu5Ac:3}	-1.937281158	FALSE	UnseenIn_File2
QDQCIYNTTYLNVQR{Hex:8; HexNAc:7; Neu5Ac:3}	-1.935589928	FALSE	UnseenIn_File2
QDQCIYNTTYLNVQR{Hex:6; HexNAc:5; Neu5Ac:3}	-1.784694778	TRUE	Passed
QDQCIYNTTYLNVQR{Hex:7; HexNAc:6; Neu5Ac:2}	-1.661444459	TRUE	Passed

QDQCIYNTTYLNVQR{Hex:7; HexNAc:6; Neu5Ac:4}	-1.613022448	TRUE	Passed
QDQCIYNTTYLNVQR{Hex:6; HexNAc:5; Neu5Ac:2}	-1.565836679	TRUE	Passed
QDQCIYNTTYLNVQR{Fuc:1; Hex:6; HexNAc:5; Neu5Ac:3}	-0.9507001545	FALSE	LowQualityIn_File2
QDQCIYNTTYLNVQR{Hex:7; HexNAc:6; Neu5Ac:3}	-0.6830903983	FALSE	LowQualityIn_File2
QDQCIYNTTYLNVQR{Fuc:1; Hex:7; HexNAc:6; Neu5Ac:4}	-0.6117663593	FALSE	LowQualityIn_File2
QDQCIYNTTYLNVQR{Hex:5; HexNAc:4; Neu5Ac:2}	-0.1850272419	FALSE	LowQualityIn_Both
QDQCIYNTTYLNVQR{@sulfate:1; Fuc:2; Hex:4; HexNAc:8; Neu5Ac:3}	NA	FALSE	BelowMinimumObs
QDQCIYNTTYLNVQR{@sulfate:1; Fuc:5; Hex:3; HexNAc:7; Neu5Ac:3}	NA	FALSE	BelowMinimumObs
QDQCIYNTTYLNVQR{@sulfate:1; Fuc:5; Hex:3; HexNAc:7; Neu5Ac:4}	NA	FALSE	BelowMinimumObs
QDQCIYNTTYLNVQR{@sulfate:1; Hex:3; HexNAc:7; Neu5Ac:3}	NA	FALSE	BelowMinimumObs
QDQCIYNTTYLNVQR{Fuc:2; Hex:7; HexNAc:6; Neu5Ac:3}	NA	FALSE	BelowMinimumObs
QDQCIYNTTYLNVQR{Hex:8; HexNAc:7; Neu5Ac:4}	NA	FALSE	BelowMinimumObs

Biological True Positive Case Studies from Prior Works

While the examples given above and in the manuscript reflect RAMZIS ability to assess highly related search results, the following RAMZIS case studies are provided to further show its utility in systems with known differentiating glycosylation. The results shown here are not identical to previously published results due to updates in the data normalization process (log-transformed abundance values are now standard), in the quality assessment processes, and in the plotting methods.

The first example looks at the Influenza A envelope protein Hemagglutinin(HA) (Uniprot: H9XJ00_I82A9) in the A/Philippines/82 strain when expressed in chicken eggs; it examines the difference between exposure the native glycosylation and the glycosylation after exposure to β 1-4 galactosidase, which removes non-reducing β 1-4 linked galactose residues from glycans. This data comes from “Measuring Site-specific Glycosylation Similarity between Influenza a Virus Variants with Statistical Certainty” by Chang et al from 2020 and shows a known biochemically induced difference. This demonstrates RAMZIS’ ability to discern biological differences in the case of disjoint glycopeptide observations.

The second example looks at HA(No applicable Uniprot Accession Number for this HA variant. FASTA Sequence provided below) from the Influenza strain A/Switzerland/9715293/2013 and the differences in glycosylation patterns between viral expression methods; one sample group comes from incubation of SWZ13 in chicken eggs (*Gallus gallus*) and the other from incubation of SWZ13 in Madin–Darby canine kidney(MDCK) cells from dogs (*Canis lupus familiaris*). There are distinct differences between the avian and mammalian glycosylation biosynthetic pathways, which will produce differences in the glycosylation patterns of the SWZ13 HA produced by them. This data comes from “Improving Statistical Certainty of Glycosylation Similarity between Influenza A Virus Variants Using Data-Independent Acquisition Mass Spectrometry” by Chang et al from 2022. This demonstrates RAMZIS’ ability to discern differences stemming from quantification and its use in planning future experimental design.

Phil82: Galactosidase’s Impact on Hemagglutinin

Galactosidase can have an extreme effect on glycosylation patterns as seen in the Chang et al 2020 paper, wherein AGP exposed to galactosidase had no overlap in glycans, but it can have more subtle effects. This was the case in Phil82 HA.

Glycans were observed at 6 N-glycosites on HA in Phil82 at the following amino acids: 38 (NATE), 144 (NNSF), 165 (NVTM), 246 (NSTG), 285 (NGSI), and 483 (NGTY). A summary of the reanalysis of all the original search results can be found in Supplemental Table 10.

Supplemental Table 10: Phil82: HA Native vs HA Gal

Glycosite	IC of Phil82 Native	IC of Phil82 Gal	Test Simulation	Overall QC	Alpha Overlap	Beta Overlap
38-NATE	2.69	2.23	PASS	PASS	0	0
144-NNSF	7.3	6.39	PASS	PASS	0	0
165-NVTM	-0.01	0.01	PASS	FAIL	0.351	0
246-NSTG	104.02	265.09	PASS	PASS*	0	0
285-NGSI	0.38	2.15	PASS	FAIL*	0.123	0.047
483-NGTY	20.69	20.52	PASS	PASS*	0	0

The IC threshold used here was a pass greater than 2, and an asterisk (*) in the Overall Quality Check status column indicates multimodality in at least one of the sample groups.

By examining the table we can see that only two sites failed to be differentiable, and those two sites failed the quality checks. These two sites were lowly glycosylated in the case of 285-NGSI, which had only 2 glycans observed in Native and 4 in Gal, and highly similar glycosylation patterns in the case of 165-NVTM, which had the same glycopeptides at similar quantifications. Both comparisons had one or both of their ICs fail to pass the threshold due to the highly similar nature of their observed quantifications. Removal of the outlier for 285-NGSI is inadvisable as there are only 3 replicates per sample group and the quality checks exist to ensure that we do not falsely reject the null-hypothesis in the Test vs Null comparison.

The biggest difference from the original analysis stems from the transition to log-normalized abundance values. 246-NSTG originally had high overlap of the Test and Null Distributions, but this is no longer the case with no overlap. The comparison passes Quality Checks as described, but this comes with a caveat: 246-NSTG Native observes a single glycan. RAMZIS sees this and recommends the user use other metrics in its output and does not provide ranking information; the comparison is pointless as the source of any differentiation is obvious.

The remaining three N-glycosites- 38-NATE, 144-NNSF, and 483-NGTY- were reconfirmed to likely have differentiable glycosylation patterns. 144-NNSF and 483-NGTY show broad differences and are heavily populated with glycans that are exclusive between sample groups. 38-NATE shows sample group exclusive glycans but is less heavily populated than the other two.

As an example, Supplemental Figure 14 shows the graphical output of the analysis of 483-NGTY from Phil 82 HA. S14A shows the relative log abundances of the two sample groups with Phil82 Native in cyan and Phil82 Gal in red; in pairs where the boxes are too small to discern color, Gal is on the left and Native on the right. S14A shows the observed glycopeptides that had at least 2 observations, which is the default minimum observation threshold for RAMZIS. S14B1 and S14B2 show the Internal Similarities of the Native (IC=20.69) and Galactosidase (IC=20.52) treated sample groups respectively. S14D shows the general comparison between the Null (blue) and Test (red) Similarity Distributions; they have no measurable overlap and the Actual Similarity has a Z-score of 0.09 in the Test Distribution indicating a successful simulation of the comparison.

Supplemental Figure 14: Phil 82 HA at 483-NGTY: Native vs Galactosidase

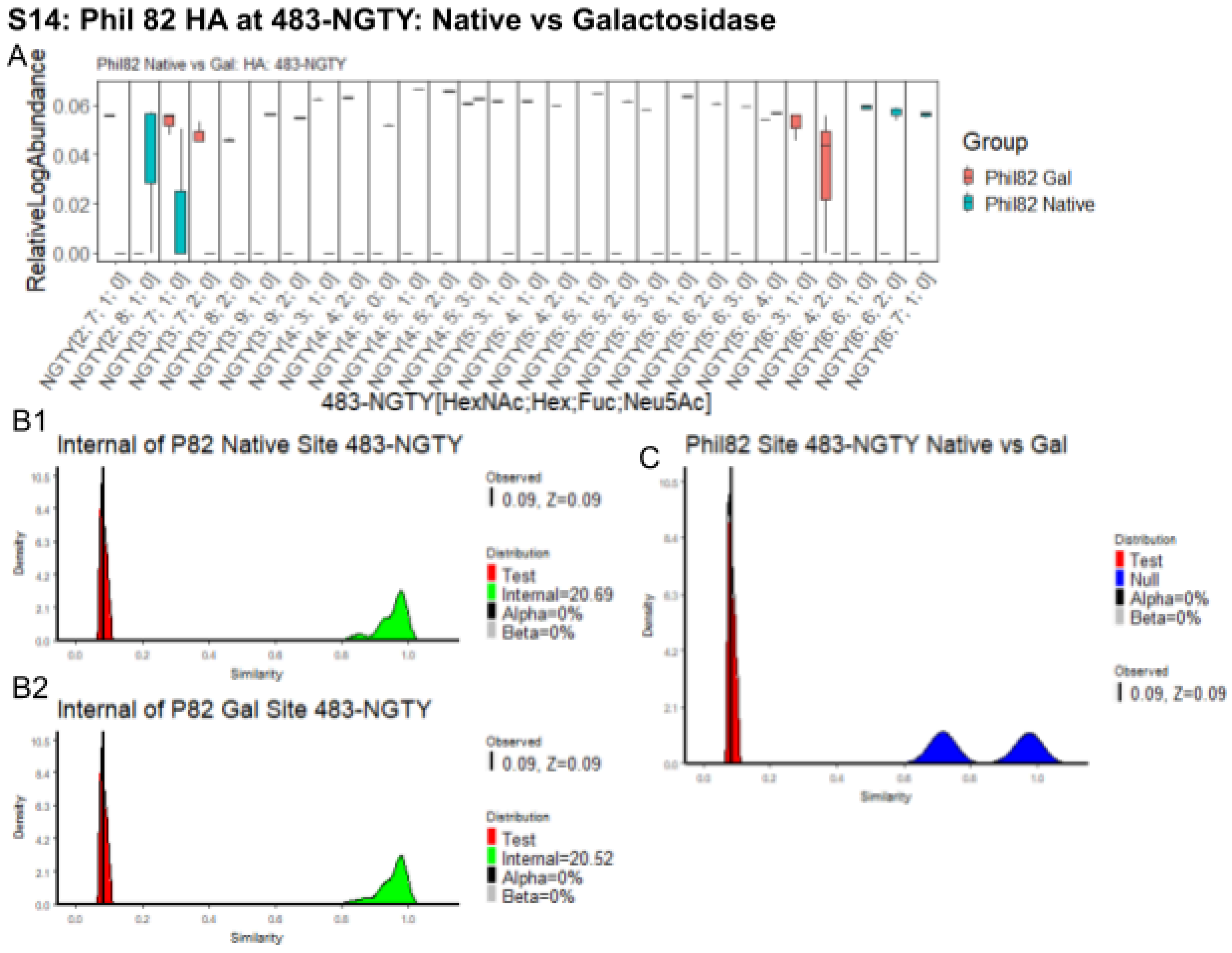

Not unexpectedly, there are a large number of exclusive glycopeptides split between the two sample groups. This comparison is obvious and easy for us to make as people, which is why it's important that RAMZIS is able to make this comparison just as easily and apply metrics to that assessment. As can be better seen in Supplemental Table 10, showing the output of the Ranking Summary provided by RAMZIS, where File 1 refers to the Native sample group and File 2 refers to the Galactosidase treated group. Rows have been colored if a glycopeptide only appears predominantly in one sample group, meaning it has one or fewer observations of the

glycopeptide in question: cyan for appearing only or predominantly in Native and red for appearing only or predominantly in Gal.

No significant contributors to dissimilarity (Z-score<-2) pass quality checks, but all of them fail due to being totally unobserved in one sample group or the other. Every single glycopeptide that is predominantly observed in only the Gal sample group has a counterpart from the Native sample group that is between 1 and 3 Hexose greater than it. This gives evidence to the hypothesis that the measurable change in the glycosylation pattern came from the galactosidase treatment.

We can also see that two glycopeptides passed their quality checks and were observed in both groups. These glycopeptides (483-NGTY[4; 5; 3; 0] and 483-NGTY[5; 6; 4; 0]) were not significant contributors to the dissimilarity observed, which we can see reflected in the relative log abundance chart in S14A. These two glycopeptides lack lower hexose counterparts in the Gal sample group, indicating that they underwent no detectable change from galactosidase. They're both highly fucosylated for the dataset, but neither has a very high number of HexNAc. Other glycopeptides with similar degrees of both can be seen to have been impacted, which indicates that something about these glycopeptides is likely different structurally. These two glycopeptides are unlikely to have reducing end hexose. Thus RAMZIS allows us to infer some structural information based on the ranking data and the relationship between glycans. We hope to expand this kind of application in future versions of RAMZIS.

Supplemental Table 10: Rankings of Phil 82 HA at 483-NGTY: Native vs Gal

SITE[HexNAc;Hex;Fuc;Neu5Ac]	ZScore	PassOverall	FailCause
NGTY[3; 8; 2; 0]	-2.05236	FALSE	UnseenIn_File1
NGTY[4; 5; 0; 0]	-2.05214	FALSE	UnseenIn_File2
NGTY[3; 9; 2; 0]	-2.05148	FALSE	UnseenIn_File2
NGTY[3; 9; 1; 0]	-2.05133	FALSE	UnseenIn_File2
NGTY[2; 7; 1; 0]	-2.05132	FALSE	UnseenIn_File1
NGTY[5; 5; 3; 0]	-2.05106	FALSE	UnseenIn_File1
NGTY[5; 6; 3; 0]	-2.05074	FALSE	UnseenIn_File2
NGTY[5; 4; 2; 0]	-2.05068	FALSE	UnseenIn_File1
NGTY[5; 6; 2; 0]	-2.05056	FALSE	UnseenIn_File2
NGTY[5; 5; 2; 0]	-2.05039	FALSE	UnseenIn_File2
NGTY[5; 3; 1; 0]	-2.05037	FALSE	UnseenIn_File1
NGTY[4; 3; 1; 0]	-2.05018	FALSE	UnseenIn_File1
NGTY[5; 4; 1; 0]	-2.05016	FALSE	UnseenIn_File1
NGTY[4; 4; 2; 0]	-2.05009	FALSE	UnseenIn_File1
NGTY[5; 6; 1; 0]	-2.05002	FALSE	UnseenIn_File2
NGTY[5; 5; 1; 0]	-2.0498	FALSE	UnseenIn_File2
NGTY[6; 7; 1; 0]	-2.04964	FALSE	UnseenIn_File2
NGTY[4; 5; 2; 0]	-2.04959	FALSE	UnseenIn_File2
NGTY[4; 5; 1; 0]	-2.04956	FALSE	UnseenIn_File2
NGTY[6; 6; 1; 0]	-2.04932	FALSE	UnseenIn_File2
NGTY[6; 6; 2; 0]	-2.04056	FALSE	UnseenIn_File2

NGTY[3; 7; 2; 0]	-2.01657	FALSE	UnseenIn_File1
NGTY[6; 3; 1; 0]	-1.99141	FALSE	UnseenIn_File1
NGTY[2; 8; 1; 0]	-0.88243	FALSE	LowQualityIn_File2
NGTY[6; 4; 2; 0]	-0.86626	FALSE	LowQualityIn_File1
NGTY[3; 7; 1; 0]	-0.53929	FALSE	LowQualityIn_File1
NGTY[5; 6; 4; 0]	-0.17559	TRUE	Passed
NGTY[4; 5; 3; 0]	-0.1545	TRUE	Passed
NGTY[3; 8; 1; 0]	NA	FALSE	BelowMinimumObs
NGTY[4; 5; 1; 1]	NA	FALSE	BelowMinimumObs
NGTY[4; 8; 1; 0]	NA	FALSE	BelowMinimumObs
NGTY[6; 7; 2; 0]	NA	FALSE	BelowMinimumObs

SWZ13: Glycosylation Differences of HA Between Chicken and Dog

It has been observed that Influenza A vaccines produced with mammalian cell derived cultures can be more effective than the more conventional method of production by chicken egg. Given that the amino acid sequence should be identical between the two vaccine production vectors, the differences in efficacy must stem from post translational modifications such as glycosylation differences. The glycan biosynthetic network of dogs to humans is more similar than that of chickens to humans; mammals have significantly less evolutionary distance to each other than they do to avians. To test this, we examined the differences in the glycosylation patterns of Hemagglutinin between the two vaccine production vectors. Hemagglutinin (HA) is an Influenza A envelope glycoprotein and is implicated in viral antigenicity and immunogenicity; it enables Influenza A to interface with host cells and infect them, and this effect has been shown to be impacted by HA glycosylation.

The reanalysis performed here is not a complete reanalysis and focuses on data not highlighted in the original manuscript. The original manuscript focused on DIA produced data, however due to concerns over current DIA glycopeptide quantification methods, we have elected to look at the DDA produced data here. This also focuses solely on the trypsin lysed samples, ignoring the chymotrypsin data, which eliminates several of the glycosites seen in the original manuscript. The glycosites seen here were 165-NVTM, 285-NGSI, and 483-NGTY.

The reanalysis found differing results from the original, but this is primarily due to the differences between the DIA and DDA data. Other factors that explain differences in results are the switch to log-transformed abundances, and a change in the relativization process that now uses a global relativization instead of a replicate specific method.

All three glycosites were found to have enough evidence to reject the null hypothesis, which is a change from the 285-NGSI DIA comparison. They had the following summary results::

165-NVTM: MDCK had an IC of 8.61; Egg had an IC of 3.56; the Actual Similarity had a Test Distribution z-score of 0.72; the Test and Null had 0 alpha and beta overlaps. MDCK had evidence of a non-impactful outlier. Two of the six glycopeptides that significantly contributed to

dissimilarity were exclusive to the Egg sample group; the remaining four had insufficient data quality in the Egg sample group to pass quality metrics.

285-NGSI: MDCK had an IC of 4.07; Egg had an IC of 5.07; the Actual Similarity had a Test Distribution z-score of 0.41; the Test and Null had 0 alpha and beta overlaps. One of the four glycopeptides that significantly contributed to dissimilarity was exclusive to the Egg sample group; the remaining three had insufficient data quality in the Egg sample group to pass quality metrics.

483-NGTY: MDCK had an IC of 7.57; Egg had an IC of 13.89; the Actual Similarity had a Test Distribution z-score of 0.076; the Test and Null had 0 alpha and beta overlaps. All glycopeptides that were significant sources of dissimilarity were sample group exclusivity.

Supplemental Figure 15: MDCK vs Egg in HA at site 165-NVTM

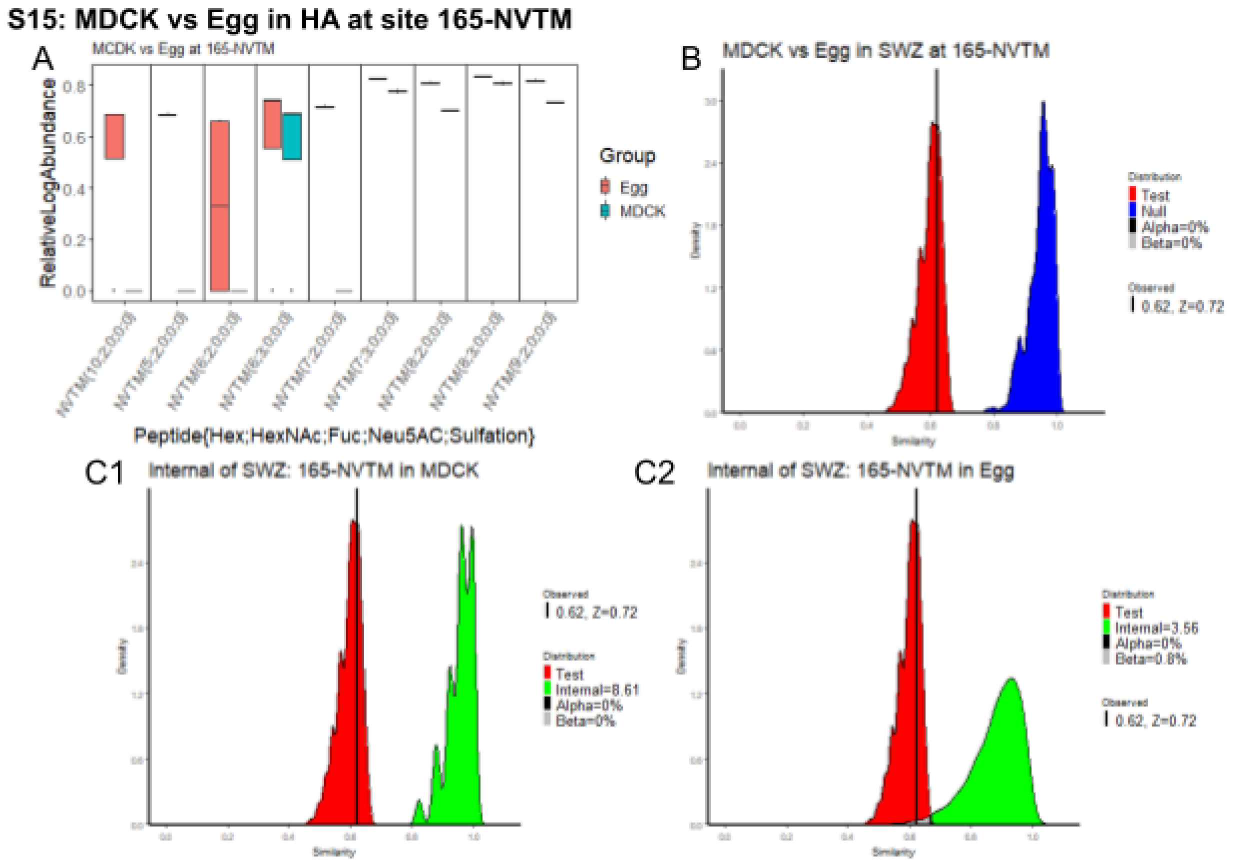

As the reanalysis of HA in Phil82 above shows a good example of differences introduced solely by exclusivity, so Supplemental Figure 15 Supplemental Table 11 will show the reanalysis of 165-NVTM. S15A shows the relative log abundances of the sample groups with Egg in red and MDCK in cyan with an n=4 for each group. S15B shows the general comparison of MDCK and Egg at site 165-NVTM with the Test Distribution in red and Null in blue; they have no overlap in the alpha or beta domains, and the Actual similarity has a Test distribution z-score of 0.72 indicating successful simulation of the comparison. S15C1 and C2 show the Internal

Distributions (green) of MDCK and Egg respectively. MDCK has an IC of 8.61 and no overlap with the Test distribution, but it does have multimodality indicating the potential presence of an outlier; as it did not impact the quality enough to be relevant to this comparison, the outlier was not removed. Egg has an IC of 3.56 and a 0.8% overlap in the beta domain, shown in grey shading. Both Internal distributions pass quality metrics of ICs greater than 2, alpha overlaps less than 5%, and beta overlaps less than 20%. This reanalysis shows evidence of a quantifiable difference in the overall glycosylation pattern at the site.

Supplemental Table 11: Ranking Summary of MDCK vs Egg at site 165-NVTM

SITE{Glycan:Composition}	ZScore	PassOverall	FailCause
NVTM{Hex:7; HexNAc:2}	-174.086	FALSE	UnseenIn_File1
NVTM{Hex:5; HexNAc:2}	-145.714	FALSE	UnseenIn_File1
NVTM{Hex:8; HexNAc:2}	-29.0619	FALSE	LowQualityIn_File2
NVTM{Hex:9; HexNAc:2}	-18.2436	FALSE	LowQualityIn_File2
NVTM{Hex:7; HexNAc:3}	-7.4003	FALSE	LowQualityIn_File2
NVTM{Hex:8; HexNAc:3}	-3.50571	FALSE	LowQualityIn_File2
NVTM{Hex:10; HexNAc:2}	-1.54825	FALSE	LowQualityIn_File1
NVTM{Hex:6; HexNAc:2}	-0.88066	FALSE	LowQualityIn_File1
NVTM{Hex:6; HexNAc:3}	0.033269	FALSE	LowQualityIn_Both
NVTM{@sulfate:1; Fuc:3; Hex:3; HexNAc:5}	NA	FALSE	BelowMinimumObs
NVTM{Hex:4; HexNAc:3}	NA	FALSE	BelowMinimumObs
NVTM{Hex:9; HexNAc:3}	NA	FALSE	BelowMinimumObs

Looking at the ranking information in Supplemental Table 11, we can see that the glycans observed all had relatively low degrees of processing with only single HexNAcs added to the branches in all but one case- which was below observational levels. The two Egg sample groups exclusive glycopeptides are colored in red. These are simple N-glycans with minimal degrees of processing, having only hexose in their branches. The remaining four glycopeptides that show significant contribution to dissimilarity all fail the quality check in the Egg sample group.

Looking at these glycopeptides relative log abundances in S15A, this quality failure may at first seem odd. These glycopeptides are shown in the four pairs on the right; they all appear to be tightly defined, low variant quantifications. And they are. But the strength of collective assessment is also a weakness; the information used to assess quality individual glycopeptides is weighted by the contributions of all glycopeptides in the sample group. RAMZIS uses a minimum observation standard of 1 glycopeptide when assessing Internal Similarity- compared to the 2 minimum observations needed for Test and Null Similarities- to be more conservative; the forced inclusion of lower reliability glycopeptides in Internal Similarity prevents false positive differentiations.

In exchange for this confidence at the global scale, it introduces a higher rate of rejection at the individual scale. In this case, the largest differential- 8 Hexose; 2 HexNAc- would need to have the MDCK log signal decreased by about 5% to be sufficiently far apart for the degree of quality available. In fact, if the minimum observation threshold for the Internal Distribution is raised to 2,

then that glycopeptide (Hex: 8; HexNAc:2) passes quality thresholds without the deleterious effects of the singly observed glycopeptides on the overall quality. Some users may find the threshold too conservative, but we should prefer certainty over positive results.

These ranking results do not even indicate a negative result; they indicate that a targeted experiment should be done on the six glycopeptides that contributed to dissimilarity. This would ensure that the glycopeptides that are absent from one sample group truly are absent, and it would give the quantitative power needed to perform more specific, less global statistical tests that would not suffer from the loss of power that a broad spectrum analysis would see in this discovery quantitation type experiment.

This reanalysis informs that there is an overall quantifiable difference and gives us targets to assess in follow up experimentation to be certain of our conclusions. The proof of a global difference provides a reasoning for exploring the niche differences and directs our attention away from sites that would benefit less from stronger quantification, such as 483-NGTY where the difference is driven by a broad array of sample group exclusive glycopeptides; there would be no point in acquiring better quantitation in a targeted experiment for those glycopeptides as the overall conclusion would not be clarified.
